# Graphene e-tattoos for unobstructive ambulatory electrodermal activity sensing on the palm enabled by heterogeneous serpentine ribbons

**DOI:** 10.1038/s41467-022-34406-2

**Published:** 2022-11-03

**Authors:** Hongwoo Jang, Kaan Sel, Eunbin Kim, Sangjun Kim, Xiangxing Yang, Seungmin Kang, Kyoung-Ho Ha, Rebecca Wang, Yifan Rao, Roozbeh Jafari, Nanshu Lu

**Affiliations:** 1 Texas Materials Institute, The University of Texas at Austin, Austin, TX 78712 USA; 2 Department of Electrical and Computer Engineering at Texas A&M University, College Station, TX 77843 USA; 3 Department of Mechanical Engineering, The University of Texas at Austin, Austin, TX 78712 USA; 4 Department of Electrical and Computer Engineering, The University of Texas at Austin, Austin, TX 78712 USA; 5 Department of Biomedical Engineering, The University of Texas at Austin, Austin, TX 78712 USA; 6 Department of Aerospace Engineering and Engineering Mechanics, The University of Texas at Austin, Austin, TX 78712 USA; 7 Department of Biomedical Engineering at Texas A&M University, College Station, TX 77843 USA; 8 Department of Computer Science and Engineering at Texas A&M University, College Station, TX 77843 USA

**Keywords:** Mechanical engineering, Biomedical engineering

## Abstract

Electrodermal activity (EDA) is a popular index of mental stress. State-of-the-art EDA sensors suffer from obstructiveness on the palm or low signal fidelity off the palm. Our previous invention of sub-micron-thin imperceptible graphene e-tattoos (GET) is ideal for unobstructive EDA sensing on the palm. However, robust electrical connection between ultrathin devices and rigid circuit boards is a long missing component for ambulatory use. To minimize the well-known strain concentration at their interfaces, we propose heterogeneous serpentine ribbons (HSPR), which refer to a GET serpentine partially overlapping with a gold serpentine without added adhesive. A fifty-fold strain reduction in HSPR vs. heterogeneous straight ribbons (HSTR) has been discovered and understood. The combination of HSPR and a soft interlayer between the GET and an EDA wristband enabled ambulatory EDA monitoring on the palm in free-living conditions. A newly developed EDA event selection policy leveraging unbiased selection of phasic events validated our GET EDA sensor against gold standards.

## Abstract (web summary)

Designing efficient sensing devices for ambulatory use remains a challenge. Here, the authors demonstrate heterogeneous serpentine ribbons enable a stretchable and robust interface between sub-micron thin graphene e-tattoos and thick and rigid printed circuit boards, which allows ambulatory electrodermal activity monitoring on the palm.

## Introduction

For decades, electrodermal activity (EDA), a.k.a. galvanic skin response (GSR), has been widely used as a quantitative index of mental stress accessible through noninvasive means^1–3^. It is gaining popularity in diverse neuroergonomic applications including psychiatry, neurology, operator and consumer assessment, as well as virtual reality and gaming^4^. Psychophysiologically, the palms are the most recommended site to monitor EDA, specifically the thenar and hypothenar eminences plus the medial and distal phalanges of the fingers^5^. It is because the palms have the highest density of eccrine sweat glands which are filled up under psychological stimuli, such as mental stress, primarily^6,7^. Commercial wearable EDA sensors connect silver/silver chloride (Ag/AgCl) gel electrodes placed on the palm to a wristband housing a rigid printed circuit board (PCB) through snap buttons and dangling wires. However, this setup poses three major problems for ambulatory EDA monitoring. First, both gel electrodes and wires on the palm are obstructive to daily activities, and also cause social stigma. Second, gel electrodes are prone to mechanical delamination from the palm during hand movements. Third, even without delamination, the electrode-to-skin impedance rises as the gel electrodes dehydrate over time, which degrades the EDA signal quality. To overcome these limitations, EDA sensors based on dry electrodes have been developed. To minimize obstructiveness, they tend to measure EDA from different locations on the body rather than the palms, including the wrists^8–11^, the forearms^12^, the shoulders^8^, and even the back^13^. However, when measured off the palms, the EDA signals can be contaminated or even interrupted by accumulative sweat secreted from the apocrine sweat glands due to the thermo-regulation of our body, an effect that is only negligible at the palmar and the plantar regions^14^. Even though a dry and wireless EDA sensor has been developed for the hand in one study^15^, its thickness of one centimeter (including circuit board and chips) makes it no less obstructive than conventional gel electrodes. Therefore, an unobstructive, long-term robust and high-fidelity palm sensor for ambulatory EDA monitoring is highly desirable but has not been available.

Ultrathin, skin-soft wearable electronics called e-tattoos have demonstrated superior skin conformability, mechanical and even optical imperceptibility, and long-term stability for monitoring various physiological signals, such as electrophysiology, skin hydration, temperature, motion, chemical biomarkers, etc^16–29^. Using large-area, monolayer graphene grown by chemical vapor deposition (CVD), our group has created sub-micron-thin graphene e-tattoos (GET), which can be highly transparent, fully skin-conformable, and can match skin stretchability when patterned into serpentine shapes^24,30,31^. Among the ultrathin wearable electrodes which can fully conform to the micro-texture of the skin, GET have demonstrated the lowest electrode-to-skin impedance and diverse sensing modalities, such as ECG, EMG, EEG, skin hydration, and blood pressure, as summarized in Supplementary Table 1^24,30–32^. However, there still exists a long-lasting challenge of how to reliably interface the sub-micron-thin, stretchable GET with millimeter-thick, rigid printed circuit board (PCB) for reliable data acquisition (DAQ) in ambulatory settings where environment is uncontrolled and skin deformation is arbitrary. Although GET is stretchable, the sub-micron thinness makes it prone to rupture even under a tiny force^33^. Therefore, conventional electrical connections for thin-film sensors such as soldering, anisotropic cohesive films (ACF), and z-axis conductive tapes are not applicable to GET. Silver paste and liquid metal have been employed to form a softer electrical contact with graphene^34,35^. However, silver paste becomes mechanically stiff once it dries. Therefore, a significant stiffness mismatch with graphene induces huge strain concentration and makes the interface fragile. Moreover, liquid phase materials need to be encapsulated by polymers, which leads to a more complex and thicker interconnect design. In fact, mechanically robust connection to rigid circuitry is a generic challenge not just limited to GET but pertinent to all types of ultrathin wearable sensors. Despite the rapid progress of sub-micron-thin, high-stretchability and skin-conformable electronics^21,23,24,32,36^, reliable and viable stretchable electrical connections to rigid DAQ system remain to be a widely recognized but unsolved bottleneck^34,37–42^. Without mechanically robust connections between ultrathin devices and rigid circuitry, those sensors cannot be practically used for ambulatory human sensing.

As a direct response to this outstanding challenge, we have constructed, tested and modeled a mechanically robust electrical contact between sub-micron-thin GET and millimeter-thick EDA wristband via heterogeneous serpentine ribbons (HSPR). In this study, HSPR refer to a GET serpentine that partially overlaps with a sub-micron-thin gold-on-polyimide (Au/PI) serpentine, both supported by the skin. Given the thinness of GET and Au/PI, all interfaces can stay adhered through just van der Waals (vdW) forces, without any added adhesives. The other end of the Au/PI ribbon is enlarged to interface with the rigid electrodes on an EDA wristband through a reusable soft interlayer with soft conductive vias. It is natural to worry about strain concentration in the GET at the Au/PI step edge, which is why only homogeneous serpentines have been used so far. But according to our finite element modeling (FEM), HSPR can lead to 50 folds of strain reduction compared to heterogeneous straight ribbons (HSTR), i.e., a straight GET ribbon partially laminated on a straight Au/PI ribbon. As a result, when HSPR is clamped end-to-end and stretched uniaxially, the overall electrical connection does not break until 42% of the tensile strain, which is similar to the stretchability of a homogeneous GET serpentine without any step edge. An equivalent circuit model with quantified components confirms that i) Au-graphene contact resistance is negligible compared with GET-skin interface impedance; ii) current flows through the GET-skin interface instead of the Au/PI-skin interface. Finally, the unobstructive GET has successfully completed a 15-hour ambulatory EDA monitoring on the palm which included studying, exercising, driving, eating and sleeping. We have also developed a novel EDA event selection policy leveraging unbiased selection of phasic events based on signal morphology, and applied it to validate our GET EDA sensor against gold standards.

## Results

### A stretchable and robust GET-wristband interface through HSPR

**Fig. 1:**
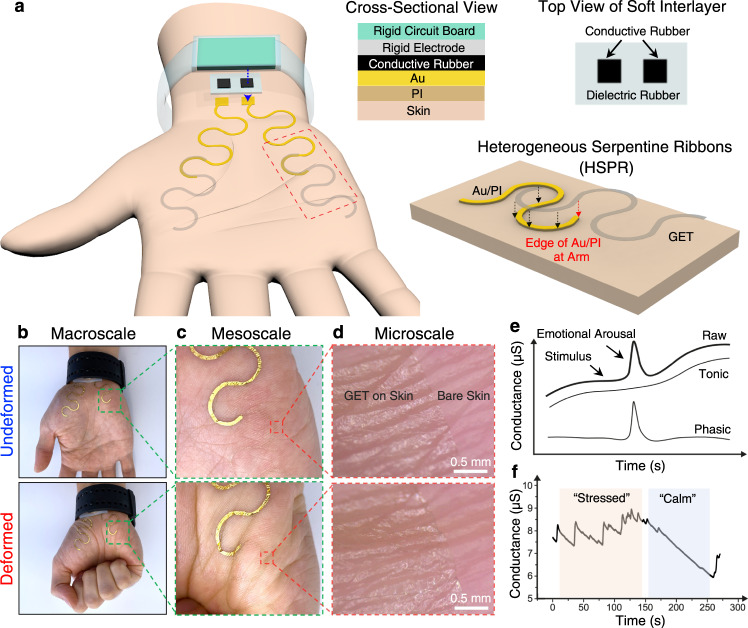
A wireless palm electrodermal activity (EDA) sensor based on sub-micron-thin graphene e-tattoo (GET) connecting to a rigid E4 EDA wristband through heterogeneous serpentine ribbons (HSPR) and a soft interlayer. **a** An overall device schematic where the detailed structures of the HSPR and the soft interlayer are illustrated in blown-up views. The HSPR is composed of GET serpentine ribbon partially overlapping with Au/PI serpentine ribbon via just van der Waals forces. The cross-sectional view along the blue dashed arrow illustrates how the Au layer is connected to the rigid electrode built into the wristband through a conductive rubber. **b**–**d** Photographs showing GET EDA sensor on the palm when the hand is undeformed and deformed in macroscale, mesoscale, and microscale, highlighting two main features of GET – transparency and skin-conformability. **e** A representative EDA signal with both low-frequency tonic component and high-frequency phasic component illustrated. **f** Example EDA signals under stressed and calm states.

Figure 1a displays a schematic of the proposed GET-wristband interface consisting of HSPR and a vertically conductive soft interlayer. All the materials in direct contact with the skin (i.e., graphene, PI, and soft silicone) are widely known as biocompatible materials^43–45^. Two GET serpentines are resting on the thenar and hypothenar eminences of the palm with graphene directly touching the skin. Each GET serpentine is partially laminated over an Au/PI serpentine ribbon with graphene touching the Au, forming the HSPR. The detailed fabrication process is described in the Methods section as well as Supplementary Figs. 1, 2. As the GET and Au/PI have similar sub-micron thinness, they can reliably laminate on the skin and with each other just through vdW interactions. An exploded view of the red dashed box highlights the overlap between GET and Au/PI. The amount of strain reduction depends on the specific location of the Au/PI step edge with respect to the GET serpentine, which will be quantified through experiment and FEM later. A silicone-based soft interlayer with two separate conductive rubber zones as illustrated in the upper right is added as a mechanical buffer layer between the Au nanomembranes and the rigid electrodes on the wristband, to prevent Au from experiencing scrubbing directly from the rigid electrodes. The cross-sectional view along the blue-dashed arrow on the wrist is expanded to illustrate the vertical lamination of different materials, starting from the rigid circuit board down to the skin.

Figures 1b to 1d are photographs of GET EDA sensor on the palm without (upper row) and with (lower row) deformation in macroscale, mesoscale, and microscale, respectively. The rigid electrodes on the back of a commercial EDA wristband (E4 wristband, Empatica Inc.) are connected to Au through the soft interlayer. The thinness of GET (300 nm) and Au/PI (750 nm) makes them mechanically imperceptible and unobstructive to the motion of the hand. Furthermore, the microscale images in Fig. 1d validate that GET is fully conformable to the microscopic surface morphology of the skin even under deformation. No adhesive is needed between any contacting layers in this construction. GET and Au/PI are strongly adhered to the skin via just vdW forces due to their ultrathinness^46^ and the high intrinsic adhesion energy (7.687 J/m^2^) between graphene and Au^47^. Moreover, our previous work has demonstrated that with the protection of liquid bandage, GET can stay on the skin for up to three days without any delamination^24^. Given the softness of the rubber interlayer, normal strapping of the EDA band on the wrist is able to secure the Au-rubber contact for ambulatory tests without any added adhesives, as proved in Supplementary Movie 3. In fact, no adhesive on the soft interlayer allows for the simple removal and reattachment of the EDA band by reusing the same soft interlayer. Figure 1e illustrates that when psychological or physiological arousal is present, the skin conductance exhibits an abrupt increase followed by a swift recovery, which is called the phasic component or the skin conductance response (SCR)^2^. The low-frequency change in skin conductance is called the tonic component or the skin conductance level (SCL), and is considered much less meaningful than SCR in stress analysis^48^. By examining the SCR signals over time, as illustrated in Fig. 1f, the mental stress level can be quantitatively determined^6,8^.

### Mechanical characterization and analysis of HSPR

**Fig. 2:**
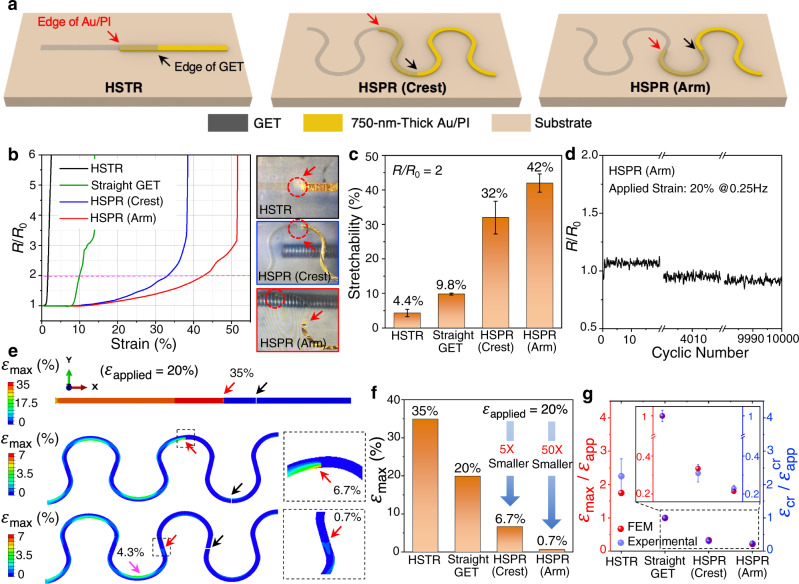
Mechanical characterization and modeling of HSPR. **a** Three different heterogeneous configurations (HSTR, HSPR (Crest), HSPR (Arm)) supported by 100-µm-thick Ecoflex substrates are stretched experimentally and numerically. The red arrow and black arrow highlight the edge of Au/PI and the edge of GET, respectively. **b** The resistance change vs. strain for the three different configurations in comparison with a homogenous straight GET. Micrographs at fracture are displayed on the right. Red-dashed circles indicate the fractured sites. **c** Stretchability extracted at *R*/*R*_0_ = 2 is plotted in a bar chart for direct comparison. Error bars represent the standard deviation of three independent tests. **d** Cyclic test of HSPR (Arm) under 20% of applied strain with 0.25 Hz up to 10,000 times. **e** FEM results of stretching HSTR, HSPR (Crest), and HSPR (Arm) without showing the Ecoflex substrate. The edge of Au/PI is zoomed in to show the strain in GET at the Au/PI step edge, which is expected to suffer from strain concentration. **f** Comparison of strains at the Au/PI step edge in the heterostructures vs. the homogenous straight GET, which confirms the strain reduction effect in HSPR, especially in HSPR (Arm). **g** Comparison between the normalized maximum strain in FEM and the normalized stretchability in an experiment for different configurations.

Mechanical characterization and analysis of HSPR are detailed in Fig. 2. Homogeneous straight GET ribbon (not drawn) and the HSTR (Fig. 2a left panel) are used as benchmarks. Two different HSPR configurations - HSPR (Crest) (Fig. 2a middle panel) and HSPR (Arm) (Fig. 2a right panel) are investigated. All ribbons are supported by 100-µm-thick Ecoflex 00-30 substrate which mimics the human skin. In Fig. 2a and the following, we always use red arrows to indicate the step edges of Au/PI and black arrows to signify the edges of GET in the heterogeneous ribbons. In this work, a 300-nm-thin GET is interfaced with a 750-nm-thin Au/PI to form HSTR and HSPR. As an electrical connector for GET, the 750-nm-thin Au/PI is chosen because it can satisfy the requirements for stretchability (higher than 45%^49^, Supplementary Fig. 3) and low stiffness mismatch^33^ (only 7.7 times stiffer than GET). In contrast, conventionally used 13-µm-thick Au/PI sheets are 37.6 times stiffer than GET, which only achieved a stretchability of 2.6% when forming a HSTR with the GET^33^. Using scanning electron microscopy (SEM), we confirm that GET can fully conform to the surface and the step edge of the Au/PI (Supplementary Fig. 4).

Another factor that can affect the stretchability of HSPR is the serpentine geometry. Our previous work has defined the unit cell of a horseshoe serpentine geometry by four parameters: the ribbon width *w*, the arc radius *r*, the arc angle $\alpha$, and the arm length *l*^50,51^. After normalization, there are three dimensionless parameters left: *w*/*r*, *l*/*r*, and $\alpha$. Some examples of serpentine shapes are displayed in Supplementary Figs. 4a-c. Our FEM results are presented in terms of the strain reduction, i.e., maximum strain in GET over applied strain (${\varepsilon }_{{\max }}/{\varepsilon }_{{app}}$), depending on *w*/*r*, *l*/*r*, and $\alpha$. We find that *w*/*r* has the largest impact on the strain reduction – almost three folds when *w*/*r* decreases from 0.8 to 0.2 (Supplementary Fig. 5d). Regarding the effects of *l*/*r* and $\alpha$, the largest strain reduction occurs when *l*/*r* is between 0 and 0.5, and $\alpha$ between 0° and 20° (Supplementary Fig. 5e, f). As skin deformation is generally considered to be around 20%^52,53^, we choose a HSPR shape with *w*/*r* = 0.2, *l*/*r* = 0.5 and $\alpha$ = 20° for the following experimental investigation. The ribbon width is fixed to *w* = 1 mm for easy laser patterning.

Figure 2b displays the end-to-end electrical resistance of ribbons supported by Ecoflex substrates normalized by its initial resistance (*R*/*R*_0_) as a function of the applied uniaxial tensile strain for different types of ribbons including an HSTR (black curve), a homogeneous straight GET (green curve), an HSPR (Crest) (blue curve) and an HSPR (Arm) (red curve). The two ends of each ribbon are fully clamped to ensure that they are fully subjected to the applied strain. The *R*/*R*_0_ are measured till the ribbons fully rupture. The fracture site is highlighted in the corresponding micrographs by red dashed circles in the right panels of Fig. 2b. As expected, fracture occurs at the Au/PI step edge for the HSTR and the HSTR (Crest). For HSPR (Arm), however, fracture is found at the crest of the GET instead of the Au/PI step edge, which implies that the crest of the GET experiences an even larger strain than the GET at the Au/PI step edge. Using *R*/*R*_0_ = 2 as a conservative criterion to quantify stretchability, the stretchability of the HSTR is found to be the lowest (4.4 ± 1.1%), followed by the homogeneous straight GET (9.8 ± 0.3%), then the HSPR (Crest) (32 ± 4.7%), and the HSPR (Arm) has the highest stretchability (42 ± 2.6%). Here, *R*/*R*_0_ = 2 is selected as the criterion of stretchability because this amount of change is equivalent to the amplitude of electrodermal activity (EDA) signals (tens of kohms) when the HSPR is applied for a wearable EDA sensor. If the inflection points of the curves in Fig. 2b, which signify catastrophic failures of the ribbons, are used to indicate stretchability, there is actually a fifty-fold enhancement in stretchability from HSTR (1%) to HSPR (51.5%), which can fully agree with the later FEM results. The stretchability results are summarized in a bar chart in Fig. 2c. The standard deviation is calculated based on three tensile tests on three different specimens of the same configuration. It is surprising that the stretchability of HSPR (Arm) (42 ± 2.6%) is similar to that of a homogenous serpentine GET (48 ± 3.4%) (Supplementary Fig. 6). This can be understood by the fact that the HSPR (Arm) has the same fracture mode as the homogeneous serpentine GET, i.e., at the crest of the GET instead of the Au/PI step edge. It suggests that the anticipated strain concentration at the Au/PI step edge (which is at the serpentine arm) did not exceed the maximum strain of a homogeneous serpentine GET at the crest. Furthermore, we validate that the bonding at the interface between GET and Au/PI via vdW forces is strong enough (i.e., no delamination or sliding under stretching) by tracking the location and shape of the trace mark near the edge of Au/PI under stretching (Supplementary Fig. 7).

The stability of HSPR (Arm) is tested through cyclic strains between 0% to 20% at a frequency of 0.25 Hz. The normalized resistance up to 10,000 cycles are plotted in Fig. 2d. The resistance even decreases slightly with the cycling, which could be attributed to the ribbon-Ecoflex interface delamination under cyclic loading and unloading.

To explain the experimentally measured stretchability of HSPR, we use FEM to simulate 20% tensile strains applied to the Ecoflex substrate and compare the strain distributions among three different configurations - HSTR, HSPR (Crest), and HSPR (Arm) - as depicted in Fig. 2e, where the Ecoflex substrate is omitted. We first confirm that any possible small gaps forming between GET and the edge of Au/PI does not affect the maximum strain on GET as long as there is no sliding in between (Supplementary Fig. 8). The maximum strains of the three configurations are plotted in a bar chart in Fig. 2f along with the homogeneous straight GET, which is the same as the applied strain, 20%. It is obvious that the maximum strain in the HSTR is the highest (35%), which is much higher than the applied strain of 20%. In contrast, the maximum strain in HSPR (Crest) (6.7%) is much less than the applied strain, which occurs at the inner crest of the GET that also coincides with the Au/PI step edge. The maximum strain in HSPR (Arm) is only 4.3% (highlighted by a pink arrow), which is smaller than that of the HSPR (Crest) (6.7%) although both occur at the crest, indicating the step edge at the crest slightly enlarges the maximum GET strain than the step edge at the arm. Surprisingly, the strain at the step edge of HSPR (Arm) is only 0.7%, which best manifests the benefit of HSPR – to locate the step edge at a strategic position of the serpentine such that the strain concentration in GET due to the step edge can be significantly alleviated. Compared with the HSTR, the strain in GET at the Au/PI step edge in HSPR (Arm) is reduced by 50 times, simply through the geometric engineering of heterogenous ribbons.

To quantitatively compare the experimental and FEM results, we borrow the following brittle fracture criterion^54^,

1 $${\varepsilon }_{{\max }}/{\varepsilon }_{{app}}\,=\,{\varepsilon }_{{cr}}/{\varepsilon }_{{app}}^{{cr}}$$

where ${\varepsilon }_{{\max }}$ represents the maximum strain calculated in FEM, ${\varepsilon }_{{app}}$ is the applied strain in FEM, ${\varepsilon }_{{cr}}$ is the critical strain-to-rupture of the straight GET measured experimentally (9.8% according to Fig. 2c), and ${\varepsilon }_{{app}}^{{cr}}$ is the experimentally determined stretchability. Equation (1) essentially offers a means to compare the FEM result (${\varepsilon }_{{\max }}$) with the experimentally measured stretchability (${\varepsilon }_{{app}}^{{cr}}$), given ${\varepsilon }_{{app}}$ = 20% and ${\varepsilon }_{{cr}}$ = 9.8% to be constants. We use ${\varepsilon }_{{cr}}$ of GET because in all the stretchability experiments, only GET ruptures, never does the Au/PI. For the four configurations, Fig. 2g plots the left of the equation (based on FEM) in red, and the right of the equation (based on experiment) in blue. The inset is a blown-up view of the black dashed box. While the FEM and experimental results are in good agreement for the straight GET, the HSPR (Crest) and the HSPR (Arm), there is a visible deviation in the case of HSTR. This deviation can be attributed to the limited experimental accuracy when the stretchability of HSTR is very small.

Although we show that HSPR especially HSPR (Arm) can significantly alleviate strain in GET under uniaxial strain, it is expected to be much less effective when subjected to transverse strain. To provide a quantitative answer, we carry out FEM with transverse stretch and the results are provided in Supplementary Fig. 9. When subjected to a transverse strain of 20% (Supplementary Fig. 9a), the maximum strain in both HSPR (Crest) and HSPR (Arm) occur at the new shallow crests with respect to the stretching direction, with very similar values (5.9% vs. 6%) as illustrated in Supplementary Fig. 7b, c, respectively. Strains at the Au/PI step edges indicated by the red arrows are only 3.9% and 5.3%, both smaller than 6%. Although the maximum strains are much higher than those appeared under longitudinal stretch, they are still ~8 times smaller than HSTR, which means the HSPR designed for longitudinal stretch still has some strain reduction effects even under transverse loading.

The effect of HSPR also depends on the stiffness mismatch between GET and Au/PI. We, therefore, apply FEM to model HSPR (Arm) involving two stiffer electrical connectors of practical use – 13-µm-thick Au/PI and 18-µm-thick Cu, which have a stiffness ratio of 37.6 and 1596 against the 300-nm-thin GET, respectively. Supplementary Fig. 10 plots the strain distributions in those two cases along with the 750-nm-thin Au/PI (Supplementary Fig. 10a) as a reference, with increasing stiffness mismatch from top to bottom. Interestingly, there is a shift of maximum strain site from the inner crest of GET serpentine to the Au/PI step edge at the arm when the stiffness mismatch becomes too high (Supplementary Fig. 10c). We also carry out the corresponding experiments and the results are summarized in Supplementary Fig. 11, which plots the stretchability dependence on the stiffness ratio, where the black markers are experimental results, and the red markers are FEM predictions based on Eq. (1). The sudden drop of the predicted stretchability for the 18-µm-thick Cu can be attributed to the change of maximum strain site. For this case, there is a larger discrepancy between the experimental and FEM results. We suspect that this is due to the buckling or delamination of the stiffer electrical connectors in the actual experiments, which is not accounted for in our FEM. Therefore, our current FEM is only applicable to HSPR with relatively small stiffness mismatches, e.g., up to 100.

### Strain isolation by soft interlayer

**Fig. 3:**
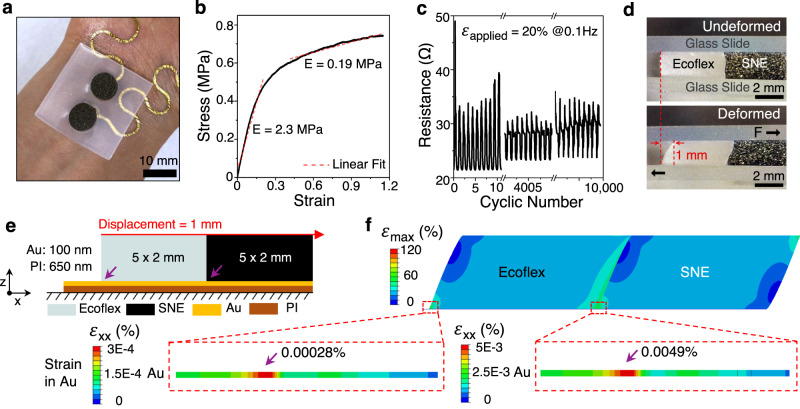
Strain isolation effect of the soft interlayer. **a** Photograph of a soft interlayer covering the Au/PI laminated on the human wrist. **b** Stress-strain curve of the commercial conductive silicone rubber (SNE-553). **c** Cyclic compression test of SNE-553 disk under 20% of compressive strain with 0.1 Hz to confirm the stability of resistance. **d** Side-view of Ecoflex and SNE-553 sandwiched by glass slides before and after a shear displacement of 1 mm. **e** FEM schematic to validate the strain isolation by the soft interlayer. Purple arrows indicate the areas of interest with potential strain concentration. **f** FEM results showing the overall strain distribution and zoomed-in strain in Au at the areas of interest. The red-dashed box highlights the strain concentration points which correspond to the two discontinuity points. Strain in the same Au/PI without the soft layer is plotted in Supplementary Fig. 12 as a comparison.

While the HSPR is very effective in limiting strains in the GET, 100-nm-thin Au on 650-nm-thin PI is also fragile. To achieve a mechanically reliable interface with rigid electrodes on the wristband, we propose to insert a soft Ecoflex interlayer embedded with two black conductive rubber disks of 8-mm diameter, as displayed in Fig. 3a, in between the Au and the wristband (Fig. 1a). The conductive rubber disks (SNE-553, Stockwell Elastomerics Inc.) are made out of silicone doped with Ni nanoparticles coated with graphite. They are tested to have low modulus (0.19 MPa - 2.3 MPa, Fig. 3b) and low vertical resistance (<50 ohms) under 20% of compressive strain, even up to 10,000 cycles (Fig. 3c). The resistance change due to cyclic compression is only about 10 ohms, which is insignificant compared to the resistance change due to EDA (tens of kohms).

Through FEM, we validate that the soft interlayer is effective in isolating the strain induced in the Au by the movement of the wristband. First, we sandwich the soft interlayer between two rigid glass slides and apply a shear force by hand to estimate an attainable shear displacement (1 mm as shown in Fig. 3d). We apply this shear displacement to our 2D cross-sectional FEM as illustrated in Fig. 3e, in which two purple arrows point to two material discontinuity points. The corresponding FEM result is plotted in Fig. 3f. The two magnified views clearly indicate the maximum strains in Au are negligibly small (0.00028% and 0.0049%) compared to the yield strain of Au (~0.2%). In contrast, without the soft interlayer (Supplementary Fig. 12), the strain induced in Au would reach 1% (fracture strain of nanocrystalline Au) under a very tiny displacement of the rigid electrode (2.5 $\mu$m).

### Electrode-to-skin interface impedance characterization

**Fig. 4:**
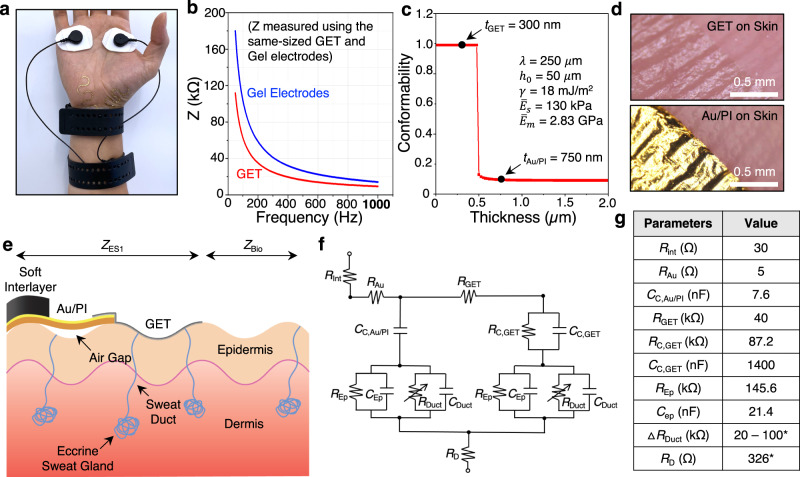
Electrode-skin interface impedance modeling and characterization. **a** A photograph of HSPR and gel electrodes connected to two identical E4 wristbands. **b** Impedance vs. frequency measured for GET (blue) and gel electrodes (red) of the same size. **c** Analytical prediction of conformability of PMMA or PI on the skin depending on its thickness. **d** Micrographs of 300-nm-thin GET (top) and 750-nm-thin Au/PI (bottom) laminated on human skin. It is obvious that only GET can fully conform to the skin, which is consistent with the analytical prediction. **e** Schematic of electrode-skin interface cross-section. Note that this drawing only displays one single electrode-to-skin interface in real EDA measurement. **f** The corresponding circuit model. **g** Values of circuit parameters either from literature (indicated by *) or from our own measurements.

Before carrying out EDA measurement with GET, we need to quantify the electrode-to-skin interface impedance because to use GET for ambulatory EDA sensing, we have added many components between the wristband and the palm skin. Figure 4a exhibits a GET-based EDA sensor and a commercial gel-based reference sensor attached to the same palm. For a fair comparison, both sensors are connected to the same type of hardware (a commercial E4 wristband) to acquire the EDA signals. It is obvious in Fig. 4a that the gel electrodes and the dangling wires are more visible and obstructive than the GET sensor. To reduce the visibility, the 750-nm-thin Au/PI could be further replaced by more transparent ultrathin interconnects such as sub-micron-thin PEDOT:PSS^22,36^. The results in Fig. 4b clearly suggest that given the same size (picture not shown), the GET could achieve lower contact impedance with the skin than the gel electrodes, which is consistent with our previous publication^24^. We attribute this phenomenon to the perfect conformability of the GET to the microscopically rough skin surface. Such conformability can be analytically confirmed using our previous mechanics models^55,56^ and GET parameters^24^. For a typical skin texture which is assumed to be sinusoidal with wavelength $\lambda$ = 250 μm and semiamplitude *h*_0_ = 50 μm, given a 2D plane strain modulus of the skin (${\bar{E}}_{{{{{{\rm{s}}}}}}}$ = 130 kPa) and a weak electrode-skin interface adhesion ($\gamma=18 \;{{{{{\rm{mJ}}}}}}/{{{{{{\rm{m}}}}}}}^{2}$), a membrane with ${\bar{E}}_{{{{{{\rm{m}}}}}}}$ = 2.83 GPa (dominated by PMMA in the GET or PI in the Au/PI) has to be thinner than 475 nm to fully conform to the skin (Fig. 4c). In fact, this is why we choose the GET to be 300 nm thin. The two micrographs of GET on skin and Au/PI on skin in Fig. 4d have confirmed this analytical prediction – the 300-nm-thin GET fully conforms to the skin whereas the 750-nm-thin Au/PI only partially conforms to the skin.

Based on the knowledge about conformability, we build a cross-sectional schematic to illustrate the complete electrode-to-skin interface. In Fig. 4e, the skin is simplified into an epidermis-dermis bilayer where eccrine sweat glands are embedded in the dermis layer and connected to the surface of the epidermis through sweat ducts. From right to left, GET fully conforms to the wavy surface of the epidermis, and Au/PI partially conforms to the epidermis and hence air gaps exist between Au and skin. The soft interlayer covers the Au and vertically connects to a rigid electrode on the E4 wristband (not drawn). According to this schematic, an equivalent circuit is built in Fig. 4f starting from the conductive soft interlayer to Au/PI, GET, epidermis, and dermis to help determine which components play a significant role in the EDA measurement. The contact resistance between GET and Au/PI is ignored because it has been confirmed to be very small (<1 ohm) when the contact area is in the mm^2^ scale^57,58^. The Au-skin interface is separated by the dielectric PI layer, so it is modeled as a contact capacitor (*C*_C, Au/PI_). In contrast, the graphene is in direct contact with the skin, so this interface is modeled to have parallel ohmic and capacitive components (*R*_C, GET_ | | *C*_C, GET_). The epidermis and dermis are represented as an RC circuit (*R*_Ep_ | |*C*_Ep_) and a resistor (*R*_D_), respectively. Finally, sweat ducts are modeled as a variable resistor parallel with a constant capacitor (*R*_Duct_ | |*C*_Duct_), and the change in the resistance ($\triangle$*R*_Duct_) due to EDA is known to be in the range of 20 – 100 kOhms^59^. The detailed measurement and calculation methods can be found in Supplementary Note 1, and the values of the parameters are listed in Fig. 4g. All capacitances are obtained at the measuring frequency of 42 Hz, which is well within the frequency range of EDA (<100 Hz). The measured *R*_Ep_ is comparable to the known reference value (100 kohms) and *R*_D_ is taken from ref. 60. The GET-skin interface resistance (*R*_C, GET_) is found to be 87.2 kohms. As the contact capacitance of GET (*C*_C, GET_) is found to be ~180 times higher than that of Au/PI (*C*_C, Au/PI_), we confirm that the Au/PI-skin contact impedance is much higher than that of the GET-skin. Thus, we conclude that the GET is the only sensing electrode of EDA in our HSPR plus soft interlayer setup. We also find that $\triangle$*R*_Duct_ is comparable to the GET-skin interface impedance which involves ${R}_{{{{{{\rm{C}}}}}},{{{{{\rm{GET}}}}}}}$ and $1/\left(\omega {C}_{{{{{{\rm{C}}}}}},{{{{{\rm{GET}}}}}}}\right)$. It actually indicates that $\triangle$*R*_Duct_ is able to make a discernable change in the overall measurement for us to successfully detect the EDA events.

### EDA measurement, event detection, and correlation analysis

**Fig. 5:**
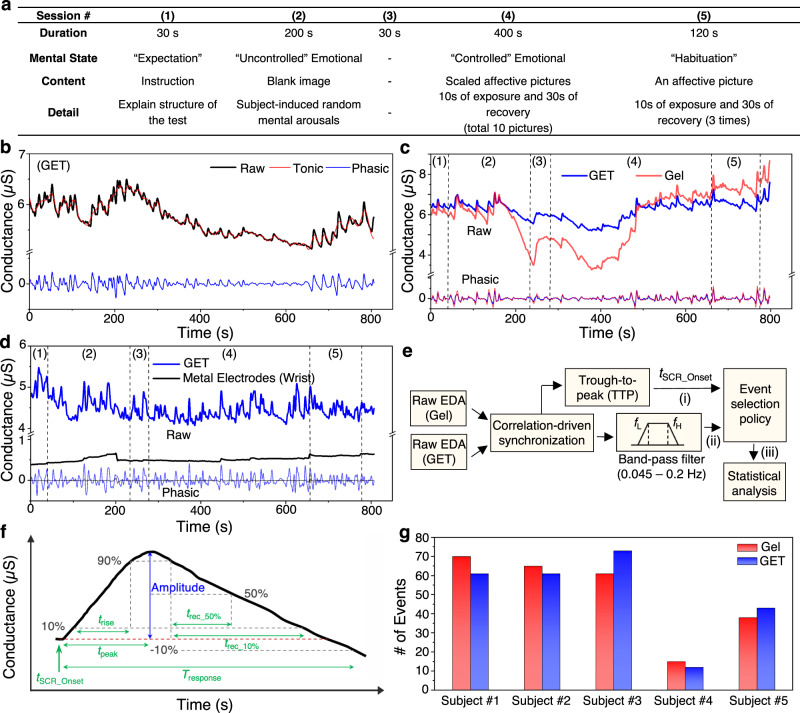
EDA measurement and correlation analysis. **a** An EDA testing video consisting of five different sessions – Expectation, “Uncontrolled” Emotional, Expectation (identical as session #1), “Controlled” Emotional, and “Habituation.” **b** Raw data and decomposed data of EDA measured by the GET sensor. **c** Comparison of EDA measured by GET (blue) vs. gel electrodes (red) on subject #1. **d** Comparison of EDA measured by GET on the palm (blue) vs. dry metal electrodes on the wrist (black). **e** SCR detection algorithm for the correlation analysis. **f** Parameters of an EDA signal used in the statistical analysis to validate the correlation between GET and gel measured EDA. **g** The total number of events measured by GET (blue) and gel electrodes (red) on five different subjects.

We measure EDA simultaneously with the GET sensor and the gel sensor for statistical comparison. To collect EDA signals from human subjects, we build a 13-minute testing video consisting of 5 different sessions as summarized in Fig. 5a. The video contains two brief introductions about the testing procedure (Sessions (1) and (3) in Fig. 5a) and three main EDA testing sessions: “uncontrolled” emotional, “controlled” emotional, and “habituation.” The details can be found in the Methods section. In this study, we carry out the EDA tests purely for the purpose of device validation instead of physiological assessment or stress level quantification.

Figure 5b plots 800 seconds of raw EDA signals measured by GET sensor (black), and its decomposition into the tonic component (red) and the phasic component (blue). Only the tonic component or SCR is considered as event-related EDA responses caused by mental or physical stress. To compare GET-measured EDA signals with gel-measured ones, a total of five EDA tests are conducted with five different human subjects (subject #1 - #5) and one representative result is given in Fig. 5c and the rest are in Supplementary Fig. 13, where blue curves are GET-measured EDA and red curves represent gel-measured EDA. In general, the GET-measured EDA has much less fluctuation in the tonic component (i.e., SCL), but is almost indistinguishable in the phasic component (i.e., SCR), compared with the gel-measured EDA. For the “habituation” session, no correlation between EDA responses and the number of repetitive exposures of the affective picture is observed. Perhaps, the random arousal from the human subjects during the “habituation” session dominates the EDA signals. We also use the rigid electrodes built in the E4 wristband to directly measure EDA from the wrist. However, no meaningful phasic components could be found, as indicated by the black curves in Fig. 5d. Therefore, only gel electrodes placed on the palm connected to the E4 wristband can obtain EDA signals comparable to that measured by our GET sensor measuring from the palm.

To statistically compare GET-measured and gel-measured EDA signals, we build a customized algorithm to detect and select good SCRs for the correlation analysis. Figure 5e provides a flow chart to illustrate our SCR selection process. As highlighted in Steps (i) and (ii) of Fig. 5e, the trough-to-peak (TTP) method is used to detect the onset of candidate SCRs for the statistical analysis. Candidate SCRs are then sent to the event selection policy (Supplementary Note 2) to count the total number of the SCRs and select only good SCRs which meet the threshold as highlighted in Step (iii) of Fig. 5e. For the SCR selection, we pick good EDA signals from gel measurement first, then compare them with the corresponding GET signals so that we don’t bias the analysis by choosing better GET signals than the gel signals. For the correlation analysis, multiple parameters of the SCR are compared, including amplitude, peak time (*t*_peak_), response time (*t*_response_), rise time (*t*_rise_), and recovery time (*t*_rec_50%_ and *t*_rec_10%_), as defined in Fig. 5f. Detailed process of the correlation analysis can be found in the Methods section. When the total number of candidate SCRs (i.e., the total number of events) are counted, as evident in Fig. 5g, the numbers of the two measurements are comparable for all five human subjects. Two datasets among the five (subjects #4 and #5) are discarded due to insufficient number of SCR satisfying the event selection policy. The calculated parameter values for the three datasets are listed in Supplementary Tables 3-5. In general, the results indicate a strong correlation of SCRs between GET-measured and gel-measured signals as the *p*-values are all greater than 0.05, i.e., the results are consistent with the null hypothesis that the EDA event parameters are equivalently captured by both types of electrodes. However, the electrode location difference on the same palm and our unbiased SCR selection algorithm can cause some of the *p* values lower than 0.05 as shown in Supplementary Tables. For all three datasets, we determine the maximum number of SCRs (N) during the “controlled” emotional session and discover that the N for the “controlled” session is very similar to the total number of applied stimulations (i.e., the number of affective pictures). We, therefore, conclude that our GET-based EDA sensor is successfully validated by the gel-based gold standards for EDA measurements.

### The wearability of GET-based EDA sensor

**Fig. 6:**
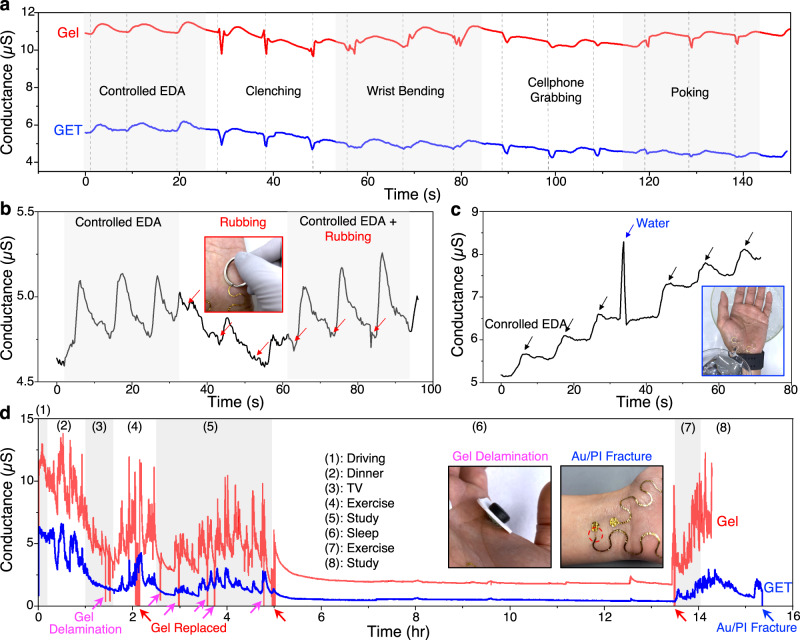
The wearability of wireless and ambulatory GET-based EDA sensor. **a** Comparison of the motion artifacts in GET- and gel-measured EDA signals when subjected to hand clenching, wrist bending, cell phone grabbing, and finger poking. **b** Rubbing GET by a metal key ring only produces negligible artifacts compared with the EDA signal. **c** EDA quickly spikes and recovers when GET undergoes a quick exposure to water. **d** Long-term, ambulatory EDA sensing using GET (blue) and gel electrodes (red) during driving, dinner, watching TV, exercise, study, sleep, exercise, and study. Gel electrodes were frequently delaminated and had to be replaced three times. Insets show the photographs of gel electrode delamination and Au/PI rupture beneath the soft interlayer.

A well-known disadvantage of dry electrodes is their susceptibility to motion artifacts. This is mainly because the contact between conventional rigid dry electrodes and skin is unsecured during motion. Figure 6a presents the EDA signals simultaneously measured by GET and gel electrodes on the palm during various types of motions. The amplitude of GET-measured conductance is found to be lower than that of the gel because the size of the exposed GET serpentine (0.6 cm^2^) is 2.5 times smaller than the size of a circular gel electrode (1.5 cm^2^). At first, three controlled EDA responses are produced without any motion by applying thermal stimulations inspired by Posada-Quintero et al.^61^. Next, different types of movements such as hand clenching, wrist bending, cellphone grabbing, and poking, are implemented three times each as indicated by the dashed lines in Fig. 6a. Comparing the two EDA responses, we could validate that despite being dry electrodes, the GET has slightly smaller motion artifacts than the gel electrodes. Also, the motion artifacts appear to have completely different morphology from the SCR signals, which means they can be easily identified and removed through either visual inspections or our event selection algorithms.

In addition to motions, we also test the performance of GET under representative incidents such as metal rubbing (Fig. 6b) and water exposure (Fig. 6c). In Fig. 6b, firstly, three controlled EDA responses are generated by applying thermal stimulations. Then, the GET is rubbed by a metal key ring three times. Lastly, three controlled EDA responses are produced while the GET was rubbed. It is evident that rubbing the GET produces a small dip in the conductance, but it has a negligible impact on the SCR signal. Furthermore, the GET-based EDA sensor is rubbed on the metallic palm rest of a laptop keyboard and a wood desk for 300 cycles each to validate its durability under repetitive friction (Supplementary Fig. 14 and Supplementary Movie 1). These two conditions are chosen because they are the objects where the palm is rubbed most often in our daily life. Our device survives 600 cycles of rubbing and can generate comparable EDA responses before and after the cyclic frictions as displayed in the inset figures of Supplementary Fig. 14. In addition, we confirm the survivability of the GET sensor under momentary exposure to water. In Fig. 6c, three controlled EDA responses are produced as benchmarks. While a spike in EDA is observed when water is poured on the GET, the conductance quickly recovers, and SCR signals can be detected again by the GET without any signal degradation. Since environmental humidity has influence on the cuticle water content and sweat gland secretion^2^, we have investigated the effect of environmental humidity on the performance of our GET-based EDA sensor. Gel-based EDA sensor is also worn on the same palm as a reference device. Supplementary Figure 15a shows a homemade humidity chamber and relative humidity (RH) is changed from 54% to 94% and recovered to 59% within 20 minutes. We find that the baseline of the measured conductance, i.e., the skin conductance level (SCL), elevates as the humidity increased and vice versa. But the controlled EDA responses, i.e., the skin conductance response (SCR), are not affected by the humidity change because SCR are only phasic responses (Supplementary Fig. 15b). The whole experimental process and the real-time data can be found in Supplementary Movie 2.

Ultimately, we demonstrate ambulatory long-term EDA sensing using our GET-based EDA sensor. Gel-based EDA sensor are used as a reference although it is uncomfortable, obstructive, stigmatizing, and unstable to wear for long-term. Figure 6d shows long-term, ambulatory EDA data taken from human subject #6 while the subject is performing a variety of daily activities. The GET sensor finishes three (see the other two in Supplementary Fig. 16) 15-hour-long nonstop ambulatory EDA measurement sessions without needing any replacement, and no human subjects report any skin irritation issues during this study. When EDA stops recording, mechanical failures are not found in the GET but in the Au/PI beneath the soft interlayer (Fig. 6d). Interestingly, the Au/PI rupture site is identical to the location of Au with the maximum strain from the shear of the soft interlayer (Fig. 3f). For subject #3 (Supplementary Fig. 16b), the so-called EDA storm during sleep^62^ is observed by the GET sensor. The gel electrodes on subject #3 fails to detect the EDA storm due to the partial delamination which happens during sleep. Unlike Fig. 6d and Supplementary Fig. 16a, Supplementary Fig. 16b shows delamination of GET which produced noises (highlighted by blue arrows). This occurs when the GET is covered by a relatively thick overlay (47-µm-thick Ecoflex). The thick Ecoflex can induce GET delamination from the skin. It emphasizes that the substrate-free design of the GET sensor is crucial to perform reliable long-term EDA monitoring on the palm with minimal noise. Additionally, to emphasize the capability of ambulatory EDA monitoring, Supplementary Movie 3 demonstrates real-time ambulatory monitoring of daily activities, such as 1) walking and running, 2) laying down, and 3) driving, and the corresponding EDA data is displayed in Supplementary Fig. 17. It clearly shows that the imperceptible GET-based EDA sensor has much less motion artifacts especially from running than the thick and obstructive gel-based EDA sensor.

## Discussions

With the emergence of ultrathin and ultrasoft electronics such as e-tattoos, stretchable and robust interfaces to thick and rigid back-end circuits become an outstanding challenge. This work introduces the first stretchable interface between sub-micron-thin devices and mm-thick rigid circuit boards. Using graphene e-tattoos (GET) connecting to a rigid wristband as an example, we apply heterogeneous serpentine ribbons (HSPR) and soft interlayer to significantly reduce the strains in graphene and Au nanomembrane. The HSPR with the Au serpentine terminating at the arm of the graphene serpentine offers the most significant strain reduction (50 folds) compared with heterogeneous straight ribbons (HSTR). Moreover, we provide a framework to design or predict the stretchability of HSPR based on finite element modeling (FEM). Our finding is surprising that HSPR (Arm) has almost the same strain releasing capability as homogeneous serpentine ribbons, despite the presence of a step edge at the arm of the serpentine. Through correlation analysis, we confirm that the GET-based EDA sensor has a similar event detection capability as the gel electrodes without motion. With motion, especially in ambulatory settings, GET-based sensors can obtain much more stable EDA signals than gel electrodes as gel electrodes are obstructive and easy to detach from the skin. The concept of HSPR is certainly not just limited to GET. The mechanics is generalizable to other ultrathin skin-conformable electronics, including MoS_2_-based touch sensors^63^, Au nanomeshes^23^, ultrathin AgNWs/PDMS^21^, PEDOT:PSS-based tattoo electrodes^36^, etc.

## Methods

### Materials

Monolayer CVD graphene grown on a copper foil is purchased from Grolltex Inc. Polyimic acid (PAA) solution and N,N-Dimethylacetamide (DMA) are purchased from Sigma-Aldrich Co. Ferric chloride (FeCl_3_) solution (CE-100) is purchased from Trancene Company Inc. Copper foil is purchased from Alfa Assar, Thermo Fisher Scientific. Chromium and gold are purchased from Kurt J. Lesker Co. Temporary tattoo paper is purchased from Silhouette America Inc. Silicone rubber (Ecoflex 00-30) is purchased from Smooth-On Inc. Conductive silicone rubber (SNE-553) is purchased from Stockwell Elastomerics Inc. Ag/AgCl based gel electrodes (EL 507) are purchased from BIOPAC Systems Inc.

### Fabrication of Au/PI electrical connector

Polyamic acid (PAA) solution is diluted with N, N-Dimethylacetamide (DMA) with a 2:1 volume ratio. The diluted PAA solution is spin-coated at 1000 rpm for 45 s on a 25-µm-thick Copper foil and pre-baked at 150 °C for 5 min and baked at 250 °C for 60 min. The copper foil is etched in ferric chloride (FeCl_3_) solution for 2 hours then transferred on a tattoo paper. To improve adhesion between Gold and a polyimide film, 5-nm-thin Chromium is deposited first on the polyimide film and then 100-nm-thin Gold is deposited.

### Fabrication of HSPR

Our group previously developed a “wet transfer, dry patterning” process to fabricate GET^24^. It involved a conventional wet transfer of large-area CVD graphene through PMMA coating and copper etching but utilized a “cut-and-paste” process^64^ to pattern the graphene/PMMA bilayer by mechanical or laser cutting to avoid chemical contaminations on graphene associated with photolithography. To fabricate HSPR, we adopt the same wet transfer process except that we switch the backing layer from PMMA to PI given the better stretchability of PI. Because the serpentine ribbons are hard to align after patterning, we decide to perform cutting on a heterogeneous sheet of Au/PI-GET where the Au/PI is strategically overlapped with GET, depending on where we want to locate the Au/PI step edge. Supplementary Fig. 1 illustrates the overall “laminate-cut-paste” fabrication process of HSPR starting from forming a 300-nm-thin PI layer over a large-area CVD graphene grown on copper. The curve of PI on graphene thickness vs. spin-coating speed is reported in Supplementary Fig. 2. After curing the PI layer, the copper foil is etched in ferric chloride (FeCl_3_) solution and the graphene/PI bilayer (Gr/PI) is rinsed in DI water. The sheet resistance of monolayer Gr on PI is measured to be 1.2 kohm/sq. To reduce the resistance, one more CVD graphene layer is added by simply laminating the Gr/PI on another CVD graphene on copper with graphene touching each other and then etching away the copper. The sheet resistance of Gr/PI is reduced 2.9 times to 410 ohm/sq by forming bilayer graphene. The bilayer graphene supported by a PI is transferred onto a commercial temporary tattoo paper with graphene facing up. Next, 750-nm-thin Au/PI bilayer (100-nm-thin Au on 650-nm-thin PI) with a cutaway is laminated on the same tattoo paper with Au facing the graphene and a partial coverage over the Gr/PI. Laser cutting of the HSPR needs to align with the Au/PI cutaway to locate the edge of the Au/PI at the arm of the Gr/PI serpentine. After the extraneous areas are removed, the HSPR on the tattoo paper can be flipped over to paste to the skin. The HSPR can be released effortlessly by wetting the backside of the tattoo paper, leaving a GET over Au/PI HSPR on the skin where the step edge is located at the arm of the GET serpentine. To locate the step edge at the crest of the GET serpentine, no cutaway in the Au/PI is needed and the edge of the Au/PI should align with the crest of the GET serpentine which is defined by the laser patterning.

### Fabrication of soft interlayer

First, a 3 cm × 3 cm × 2 mm (L × W × H) mold for the soft interlayer is built using a 3D printer (MakerBot, MakerBot Industries LLC.). Then, Ecoflex is poured into the mold and cured at 60 °C for 3 hours. In the meantime, a sheet of conductive silicone rubber (SNE-553) is cut into circles with a diameter of 8 mm. After the Ecoflex is cured, it is removed from the mold and two holes with 8 mm diameter are punched. Then, the circle-shaped conductive silicone rubber is inserted into the Ecoflex matrix.

### Mechanical characterization of HSPR

A customized low-profile stretcher is used to analyze the electromechanical behavior of HSPR. The stretcher is integrated with a 1 RPM gear motor (TS-32GZ370-5300, Tsiny). The HSPR is clamped at both ends with an Au/PET (100-nm-thick Au on 13-µm-thick PET) ribbon. The clamps of the stretcher are encapsulated by double-sided tape. The Au/PET connectors are connected to NI ELVIS II (National Instruments Educational Laboratory Virtual Instrumentation Suite) via alligator clips. The change of resistance is recorded in situ via NI LabVIEW with a sampling frequency of 10 Hz while the HSPR is stretched. The cyclic test is performed using a dynamic mechanical analyzer (RSA-G2, TA Instruments.) with the same clamping method as above to acquire resistance.

### Mechanical characterization of soft interlayer

To measure the modulus of conductive silicone rubber (SNE-553), the SNE-553 is cut into a 20 mm × 3 mm (gauge length x width) ribbon. A dynamic mechanical analyzer (RSA-G2, TA Instruments.) is used to apply a tensile strain up to 120% on the SNE-553 ribbon and a stress-strain curve is obtained. The same dynamic mechanical analyzer is also used to perform the cyclic test and the resistance is measured in situ using NI ELVIS II and NI LabVIEW with a sampling frequency of 5 Hz.

### Finite element modeling of HSPR and soft interlayer

We carry out finite element method (FEM) simulation of HSPR and soft interlayer using commercial software ABAQUS (standard 6.13). Dynamic implicit step with nonlinear geometry is implemented. The HSPR and the stretching substrate (100-µm-thick Ecoflex) are modeled using a 3D deformable shell with S4R elements. Each layer is partitioned accordingly and assumed that there is no delamination at the interface. For GET, 300-nm-thin PI is assigned as an elastic material with a modulus of 2.5 GPa and Poisson’s ratio of 0.34. For Au/PI, 100-nm-thin Au is assigned as an elastic material with a modulus of 79 GPa and Poisson’s ratio of 0.42, and 650-nm-thin PI is assigned with the same PI property mentioned above. Ecoflex is modeled as an incompressible Neo-Hookean hyperelastic material with a modulus of 0.1 MPa. The HSPR is stretched end-to-end with 20% of applied strain and no out-of-plane deformation is allowed.

For FEM simulation of the soft interlayer, a general static step with nonlinear geometry is implemented. Plane strain condition is assumed so only 2D cross-section needs to be modeled, as shown in Fig. 3e. Ecoflex, SNE-553, and PI are modeled as 2D deformable planes and Au is modeled as a 1D beam. Ecoflex is modeled as a nearly incompressible Neo-Hookean hyperelastic material with a modulus of 0.1 MPa and Poisson’s ratio of 0.475. SNE-553 is modeled as a nearly incompressible Neo-Hookean hyperelastic material with a modulus of 2.2 MPa and Poisson’s ratio of 0.475. PI is modeled the same as described in the section above. The bottom of PI is fixed, and a shear displacement of 1 mm is applied on the top edge of Ecoflex and SNE-553 to find the strain distribution on the structure due to the shear lag.

### Electrode-to-skin interface characterization

Using a HIOKI 3532-50 LCR meter, we measure the electrode-to-skin impedance from 42 Hz to 1000 Hz, using rectangle GET and circular gel electrodes of the same size (1.5 cm^2^) contacting the skin. Detailed methodology to find parameters of the equivalent circuit can be found in Supplementary Note 1.

### Integration method of GET-based EDA sensor on the palm

After HSPR is transferred onto the skin, a soft interlayer is mounted on top of the Au/PI by aligning the conductive pads (SNE-553) with the Au terminals. The rigid electrodes of the wristband (E4 wristband, Empatica Inc.) are aligned with the conductive pads in the soft interlayer. Finally, the wristband wraps the wrist with comfortable pressure.

### EDA measurements and statistical data analysis

EDA testing video is taken and processed using commercial software (DaVinci Resolve 17, Blackmagic Design Pty. Ltd.). Skin conductance is measured using GET and gel electrodes on the palm and the measured data is transferred to the mobile device and saved in a Cloud drive via the commercial wearable wristband (E4 wristband, Empatica Inc.) with a sampling frequency of 4 Hz. For the dry metal electrode measurements, the Ag-plated dry EDA electrodes from Empatica Inc. are used to measure the skin conductance on the wrist. For human subject testing, six healthy men and women in the age of 25-35 (5 males and 1 female) volunteer to participate. Human subjects watch the testing video in a quiet room alone while wearing both GET and gel sensors on the same palm. During the 200-second “uncontrolled” emotional session, the human subjects are presented with a blank screen, so their thoughts are unaffected, which results in random mental arousals. During the 400-second “controlled” emotional session, the participants are presented with a series of scaled affective pictures. Those scaled affective pictures are taken from the EmoMadrid database^65^ and the details about each picture used are listed in Supplementary Table 2. Finally, during the “habituation” session, a single affective picture is shown three times to study whether the arousal level decreases as the number of repetitive exposure increases.

We process the raw EDA readings simultaneously captured by gel and GET electrodes using MATLAB (The MathWorks, Inc.) to perform statistical analysis. To eliminate possible time delay between different sensors, we examine the cross-correlation between gel and GET measured raw EDA signals at different time shifts and synchronize the two signals using the time shift that gives the highest correlation value. We apply a 6th order Butterworth low-pass filter at 0.2 Hz to mitigate high-frequency noise from the synchronized signals. To differentiate the phasic and tonic EDA components from the full EDA waveform, we apply a 4th order Butterworth at 0.045 Hz cut-off frequency as high-pass and low-pass filters, respectively. To detect any candidate skin conductance response (SCR), we apply the trough-to-peak (TTP) method to the filtered EDA signals that mark all instances of local minima and maxima using Ledalab software^48^. We select a minimum of 0.05 $\mu$S SCR threshold amplitude to avoid incorrect measurements due to motion artifacts and other noise contributions. The output of the Ledalab marks the time values of all candidate SCR event onsets. This onset timing information is used in conjunction with the phasic EDA signals to identify SCR events that will be used in the statistical analysis. The identification of the useful events is through an event selection policy that goes over all candidate events and eliminates events that do not comply with the predetermined policy (Supplementary Note 2). Mean error (ME), 95% confidence intervals of ME, and t-test *p*-values are calculated for each feature to find the statistical correlation between Gel and GET EDA signals.

### Wearability test of GET-based EDA sensor

For all wearability tests, commercial liquid bandage (Nexcare liquid bandage spray, 3 M) is sprayed over the GET and Au/PI after they are transferred and conformed on the palm to form a μm-thin, transparent solid protection layer. To provide controlled EDA responses, the human subject is either pinched or thermally stimulated depending on the reactivity of the EDA responses. The thermal stimulation is applied by placing the other hand different from the EDA measurement on a hot plate of 55 °C for 1 second. For the scrubbing test, the applied pressure is measured using a flexible hybrid-response pressure sensor (HRPS)^66^ and the skin conductance is measured by the GET-based EDA sensor while the human subject manually rubbed his palm on the metallic palm rest of the keyboard and the wood desk. During the motion artifact tests, humidity test, and ambulatory long-term wearability tests, human subjects wear two E4 watches on the same wrist to save the EDA data from GET and gel electrodes. For the ambulatory long-term wearability test, the skin conductance is measured continuously throughout the day and night with daily activities such as driving, eating, watching TV, exercising, studying, and sleeping.

## Supplementary information

Supplementary Information

Description of Additional Supplementary Files

Supplementary Movie 1

Supplementary Movie 2

Supplementary Movie 3

## Data Availability

All data in this work are presented in the main text and the supplementary information.

## Code availability

The code for the EDA event selection is available from the corresponding author on reasonable request.
