# Supplementary material for: Graphene e-tattoos for unobstructive ambulatory electrodermal activity sensing on the palm enabled by heterogeneous serpentine ribbons
Source: Nat Commun. 2022 Nov 3;13:6604. doi: 10.1038/s41467-022-34406-2 (PMC9633646; doi:10.1038/s41467-022-34406-2)
Supplement: Supplementary file 1 — Supplementary Information [file 41467_2022_34406_MOESM1_ESM.pdf]

Supplementary Information for

Graphene E-Tattoos for Unobstructive Ambulatory Electrodermal Activity Sensing on the Palm Enabled by Heterogeneous Serpentine Ribbons

Hongwoo Jang, Kaan Sel, Eunbin Kim, Sangjun Kim, Xiangxing Yang, Seungmin Kang, Kyoung-Ho Ha, Rebecca Wang, Yifan Rao, Roozbeh Jafari, and Nanshu Lu*

*Corresponding author. Email: nanshulu@utexas.edu

Supplementary Note 1.

Parameter calculations in the equivalent circuit model

We used two electrodes to measure skin conductance through the skin and assume these two electrodes are identical. Also, the impedance through the deep skin or dermis is negligibly smaller than the electrode-to-skin impedance. Therefore, we can define the total impedance as

$$| Z_{\text{total}} | = |Z_{\text{ES1}} + Z_{\text{Bio}} + Z_{\text{ES2}} | = | Z_{\text{ES1}} + Z_{\text{ES2}} | = | 2Z_{\text{ES1}} |,$$

where Z_{total} means the total impedance, Z_{Bio} denotes impedance of dermis, Z_{ES1} and Z_{ES2} indicate electrode-to-skin impedance for electrode 1 and electrode 2, respectively. To find the parameters of the equivalent circuit model as shown in Fig. 3F, we first simplify the circuit model for epidermis and dermis as an equivalent RC circuit to find the R_{Ep} and C_{Ep} using two Ag/AgCl gel electrodes. Now the simplified circuit model looks as shown below.

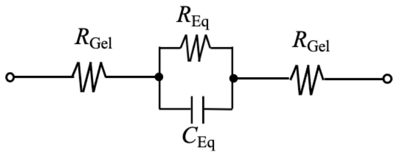

First, to find R_{Gel}, an infinitely high frequency (we used 1MHz) was applied, and the impedance was measured. Second, to find the R_{Ep}, DC (0Hz) was applied, and initial impedance was recorded to avoid the impact of the polarization. Finally, a frequency in the working range of EDA measurement (<100Hz, we used 42Hz) was applied to find C_{Ep} by using the following equation,

$$|Z_{\text{total}}| = 2 * R_{\text{Gel}} + \frac{1}{\sqrt{\left(\frac{1}{R_{\text{Ep}}}\right)^2 + \left(2\pi f C_{\text{Ep}}\right)^2}}$$

where f is the applied frequency (42 Hz). After R_{Ep} and C_{Ep} were found, Au/PI electrode was applied on the same location of skin and now the equivalent circuit becomes as follows.

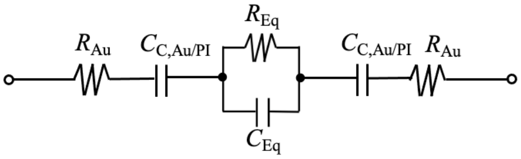

To find $C_{\mathrm{C,\ Au/PI}}$, we applied 42 Hz and measured the total impedance. Then, we used the following equation to deduce the value of $C_{\mathrm{C,\ Au/PI}}$.

$$|Z_{\mathrm{total}}| = 2 * R_{\mathrm{Au}} + 2/\sqrt{\left(2\pi f C_{\mathrm{C,Au/PI}}\right)^2} + \frac{1}{\sqrt{\left(\frac{1}{R_{\mathrm{Eq}}}\right)^2 + \left(2\pi f C_{\mathrm{Eq}}\right)^2}}$$

where f is the applied frequency (42 Hz). Finally, GET is laminated on the skin to form HSPR and now the equivalent circuit becomes as follows.

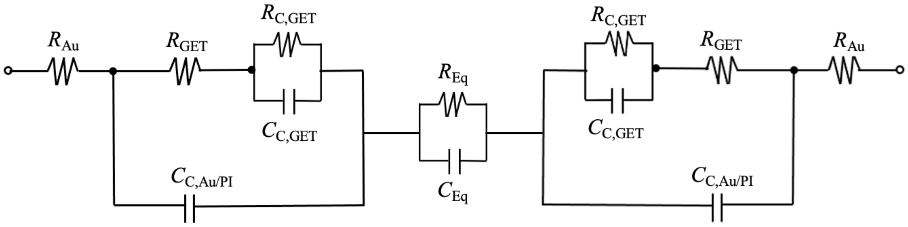

Same as the first step to find the R_{Ep} and C_{Ep}, DC (0 Hz) was first applied to find the $R_{\mathrm{C,\ GET}}$, and 42 Hz was applied to find the $C_{\mathrm{C,\ GET}}$.

Supplementary Note 2.

The event selection policy for SCR detection algorithm

Input: Synchronized and band-pass filtered EDA readings from the gel and GET $v(t) = [SCR_{gel}(t),\ SCR_{get}(t)]$, and the SCR event locations extracted with TTP, $t_{SCR\_Onset}[j]$.

Output: clean marked SCR events, $SCRc\{t, j\}$

1. *for j = 1* to τ do: // segmentation, τ = # of detected candidate events with TTP
2. $v_{segmented}[t, j] = v(t)$ where $v(t) \geq t_{SCR\_Onset}[j]$ and $v(t) < t_{SCR\_Onset}[j + 1]$
3. $\Delta SCR[j]$ =max $v_{segmented}[t, j]\ - v_{segmented}[1, j]$ // amplitude of each candidate event
4. ΔSCR_{max} = *95th percentile of* ΔSCR // maximum SCR amplitude detected
5. *for j = 1* to τ do: // process each candidate event
6. $flag[j] = 0$; // initiate the flag array, 0 means a clean event
7. if the duration of $v_{segmented}[t, j]$ < *2sec, then* $flag[j] = 1$*;* // too short for a clean event
8. if $\Delta SCR[j] < 0.1 \cdot \Delta SCR_{max}$, then $flag[j] = 1$*;* // not enough SCR amplitude
9. $v_{rise}[t, j] = v_{segmented}[1{:}\, i_{max}, j]$, where i_{max} is the timestamp index for the maximum of SCR events.
10. $v_{recovery}[t, j] = v_{segmented}[i_{max}{:}\, end, j]$, where end is the last timestamp index for the SCR event.
11. if min $v_{recovery}\,[t, j] > 0.1 \cdot \Delta SCR[j] + v_{rise}[1, j]$, then $flag[j] = 1$*;* // response did not recover more than 90% of the initial level before the next SCR event

12. if $flag[j] == 0$, then $SCRc\{t,j\} = append(v_{rise}[t,j], v_{recovery}[1: i_{10p-down,j}])$ // mark the event as a clean event, where trim down the part of the recovery that drops below 10% of the initial SCR location.

13. if $flag[j] == 1$, then $SCRc\{t,j\}$ = {} // exclude the event from further analysis

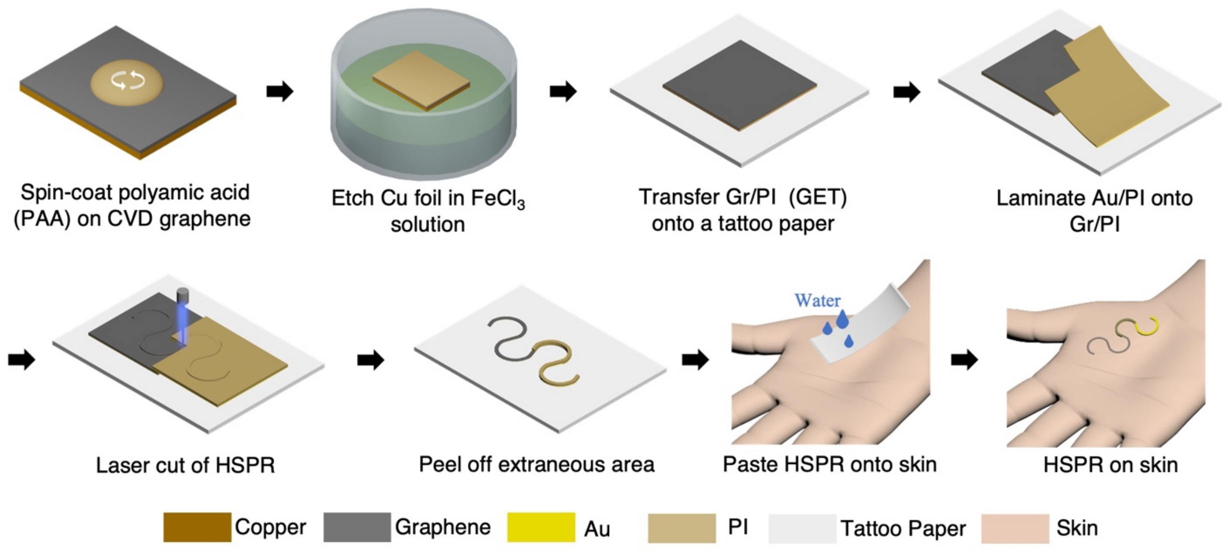

Supplementary Fig. 1. Laminate-cut-paste fabrication of HSPR (Arm). Polyimide (PI) precursor – polyamic acid (PAA) is spin-coated on a CVD graphene grown on a Cu foil. The Cu is etched in $FeCl_3$ solution and the PI-supported graphene (Gr/PI) is transferred onto a commercial tattoo paper with graphene facing up. 100-nm-thin Au deposited on 650-nm-thin PI is partially laminated over the GET. A UV laser carves the two sheets into HSPR (Arm) which indicates that the Au/PI terminates at the arm of the serpentine. The patterned HSPR (Arm) can be pasted onto human skin by wetting the tattoo paper.

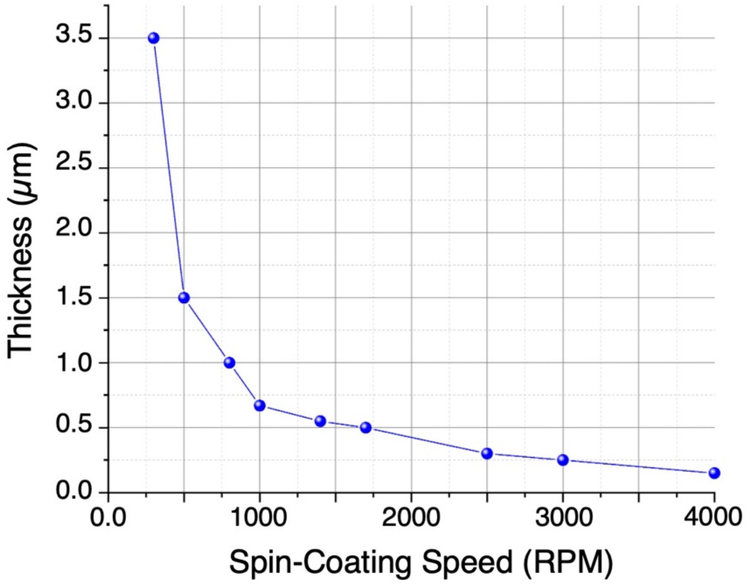

Supplementary Fig. 2. The thickness of PI depending on spin-coating speed.

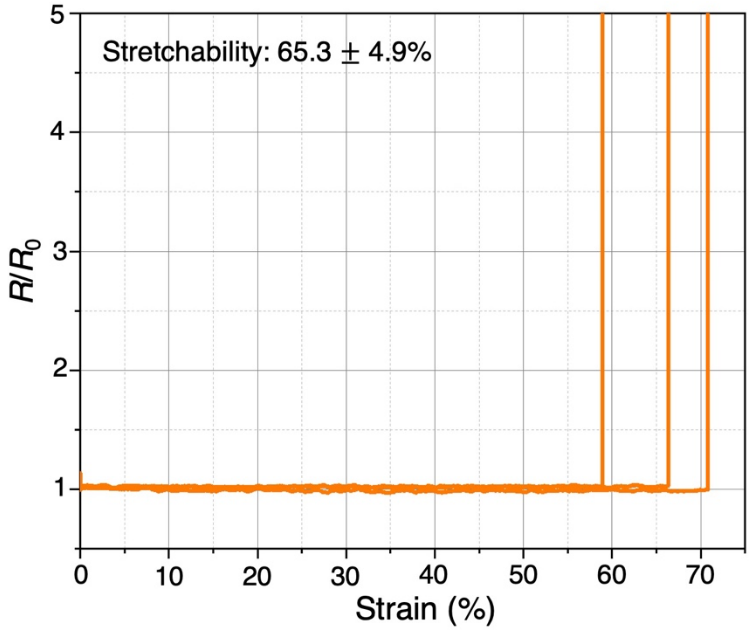

Supplementary Fig. 3. Stretchability of 750-nm-thin Au/PI. Each orange line indicates a different experimental trial.

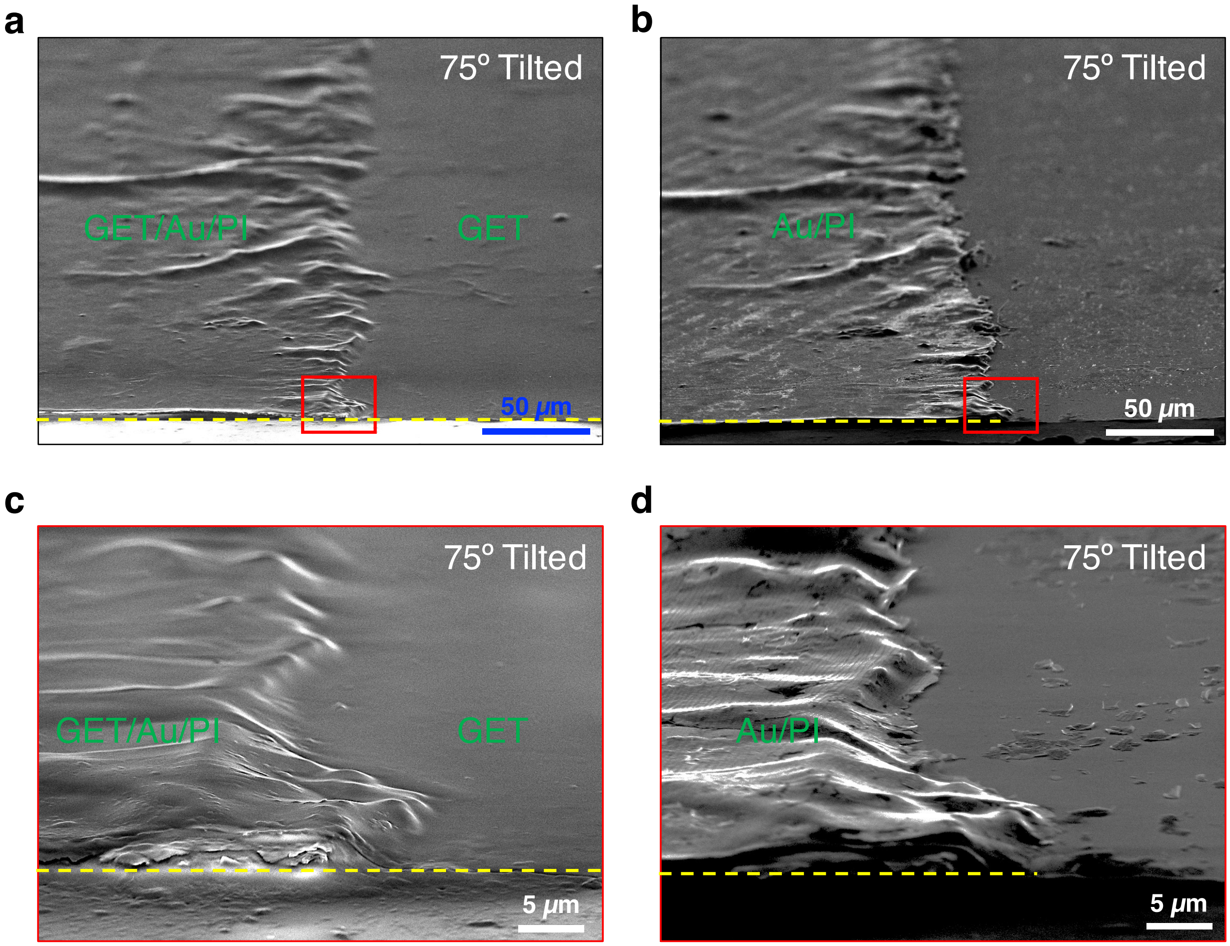

Supplementary Fig. 4. SEM micrographs of the edge of Au/PI **a)** with GET and **b)** After GET is removed. **c), d)** Zoomed-in views of the red boxes in **a)** and **b)**, respectively. The horizontal yellow dashed line indicates the surface of the supporting Si wafer.

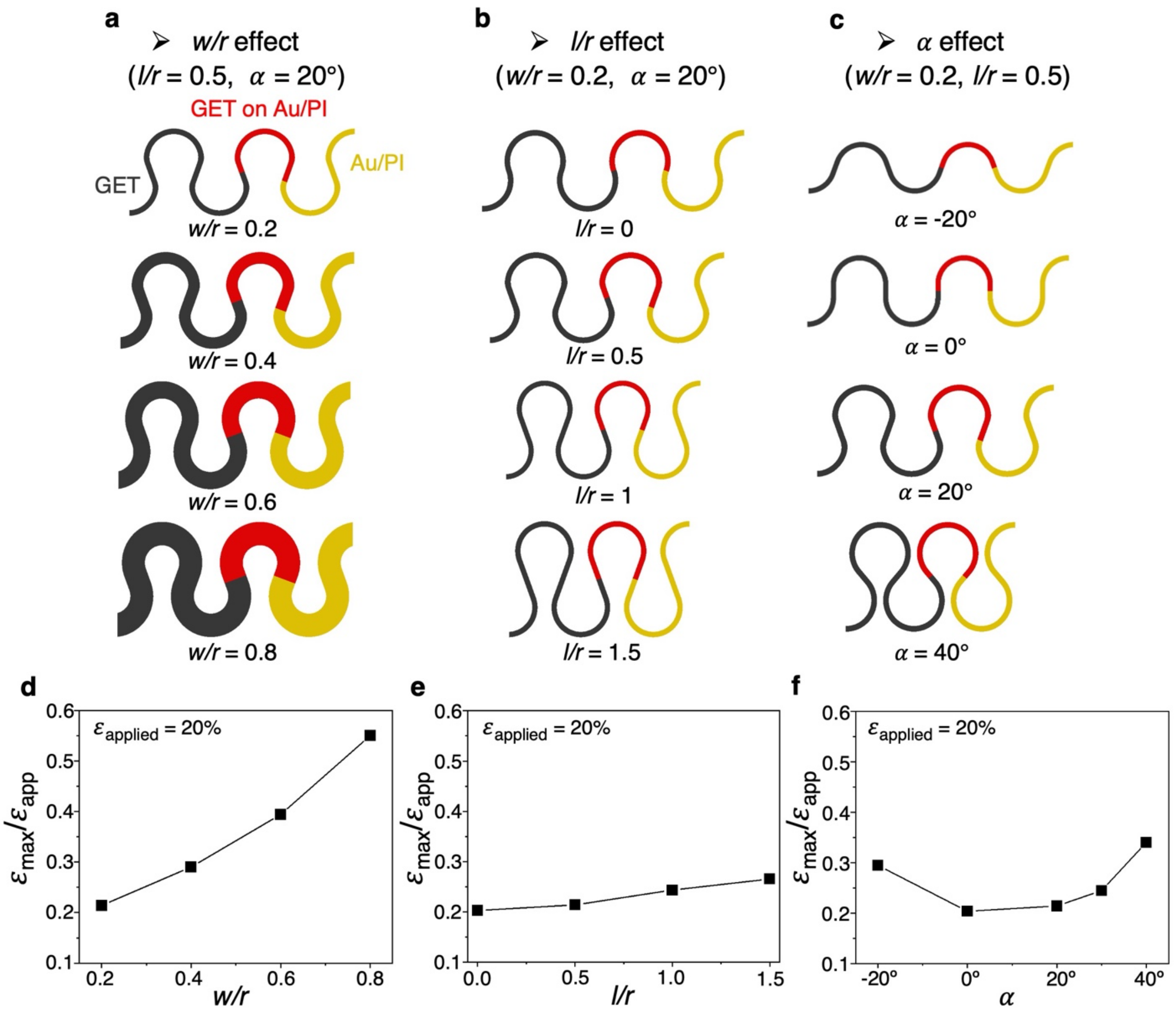

Supplementary Fig. 5. Parametric study of HSPR (Arm) geometry. a-c) Schematics of HSPR (Arm) geometry depending on **a)** *w/r*, **b)** *l/r*, and **c)** $\boldsymbol{\alpha}$. Dark grey, red, and yellow indicates GET, GET on Au/PI, and Au/PI, respectively. Strain reduction of HSPR (Arm) depending on **d)** *w/r*, **e)** *l/r*, and **f)** $\boldsymbol{\alpha}$, accordingly.

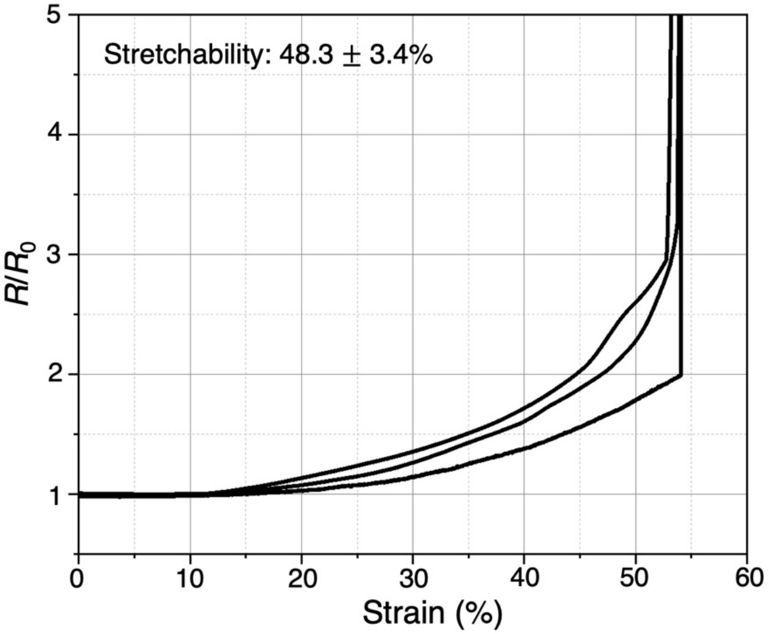

Supplementary Fig. 6. The stretchability of 300-nm-thin serpentine GET. Each black line indicates a different experimental trial.

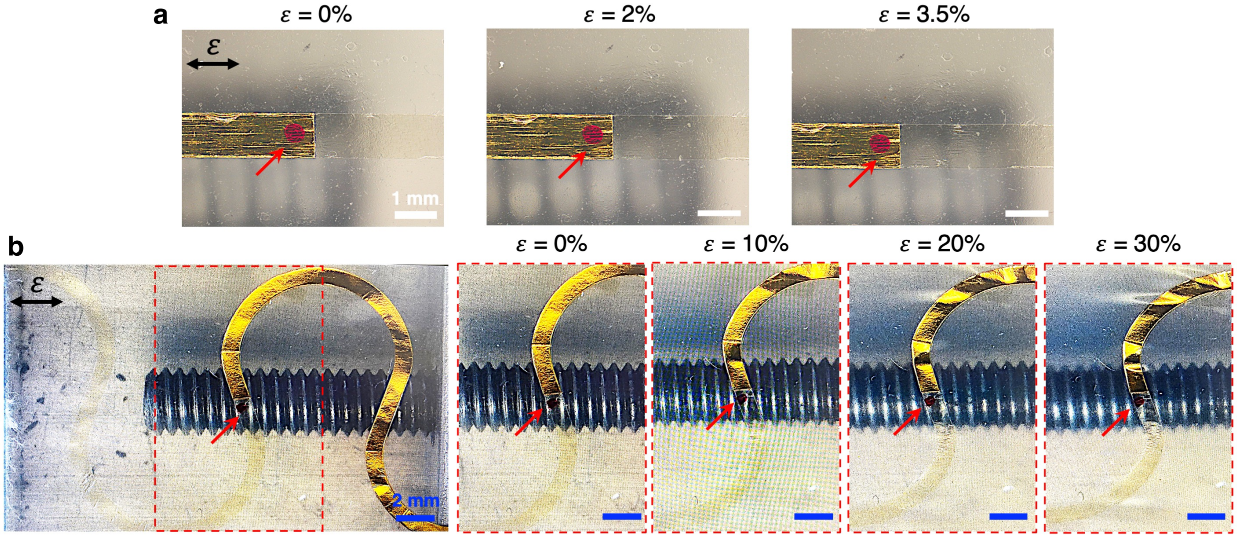

Supplementary Fig. 7. Optical micrographs of HSTR and HSPR at the edge of Au/PI. A red ink droplet is applied on the GET near the edge of Au/PI of **a)** HSTR and **b)** HSPR to track the change of its location and shape under stretching. The red arrow indicates the location of the red mark. The fact that the distance between the marker and the Au/PI edge does not change indicates that there is no slippage between GET and Au/PI after been stretched by 30%.

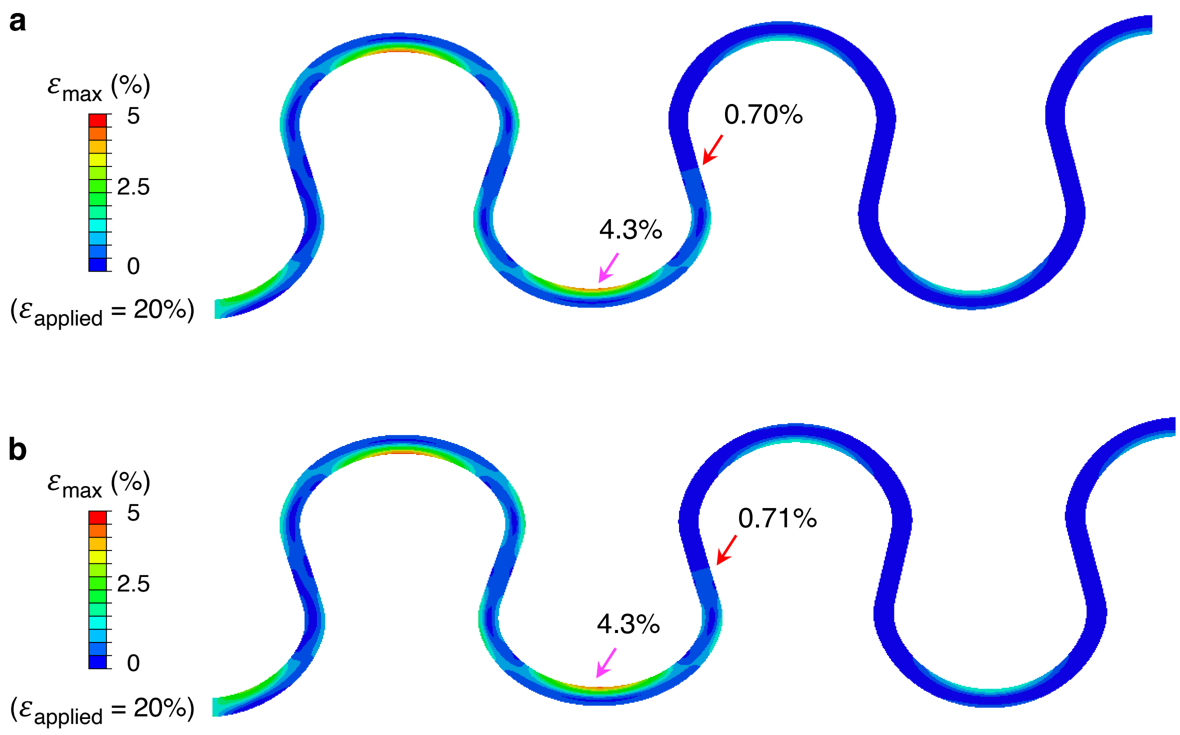

Supplementary Fig. 8. FEM investigation of the effect of an edge gap on the maximum strain in HSPR (Arm). HSPR **a)** without and **b)** with a small gap between GET and the edge of Au/PI under 20% of end-to-end longitudinal strain. The gap is modeled as a small section (25 μm) of freestanding GET (i.e., without the 100-μm-thick Ecoflex substrate). All other regions are supported by the substrate although it is not displayed in the contour plots. Red arrows indicate the edge of Au/PI and pink arrows indicate the global maximum strain. Based on this FEM result, we conclude that the edge gap does not affect the maximums strain in GET.

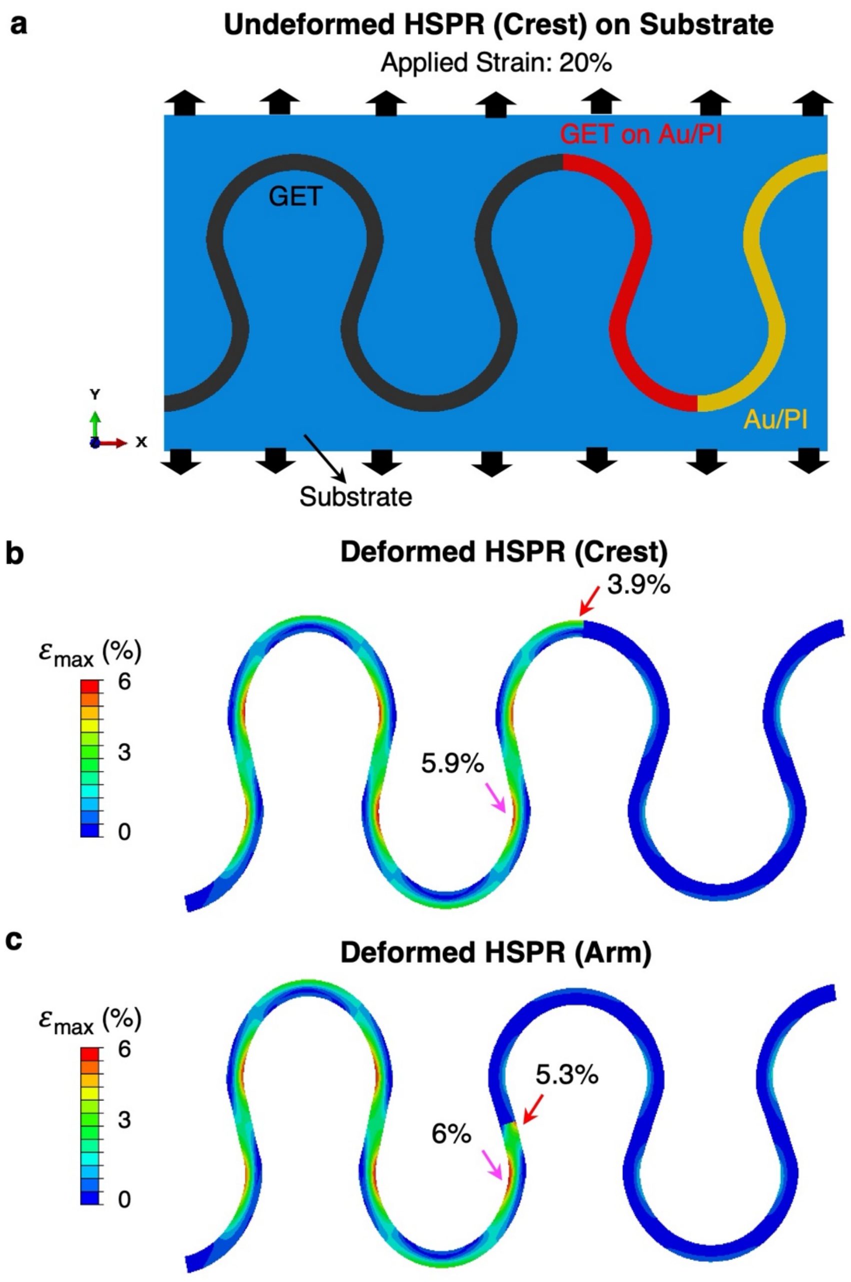

Supplementary Fig. 9. Transverse stretching of HSPR in FEM. a) 300-nm-thin GET is connected to 750-nm-thin Au/PI on a 100-µm-thick Ecoflex. 20% of strain is applied in the transverse direction. The FEM simulation results with the edge of contact of Au/PI located at **b)** crest and **c)** arm of the serpentine are displayed. The pink arrow indicates the global maximum strain, and the red arrow indicates the local maximum strain at the edge of the interface. Ecoflex is not displayed in the simulation results.

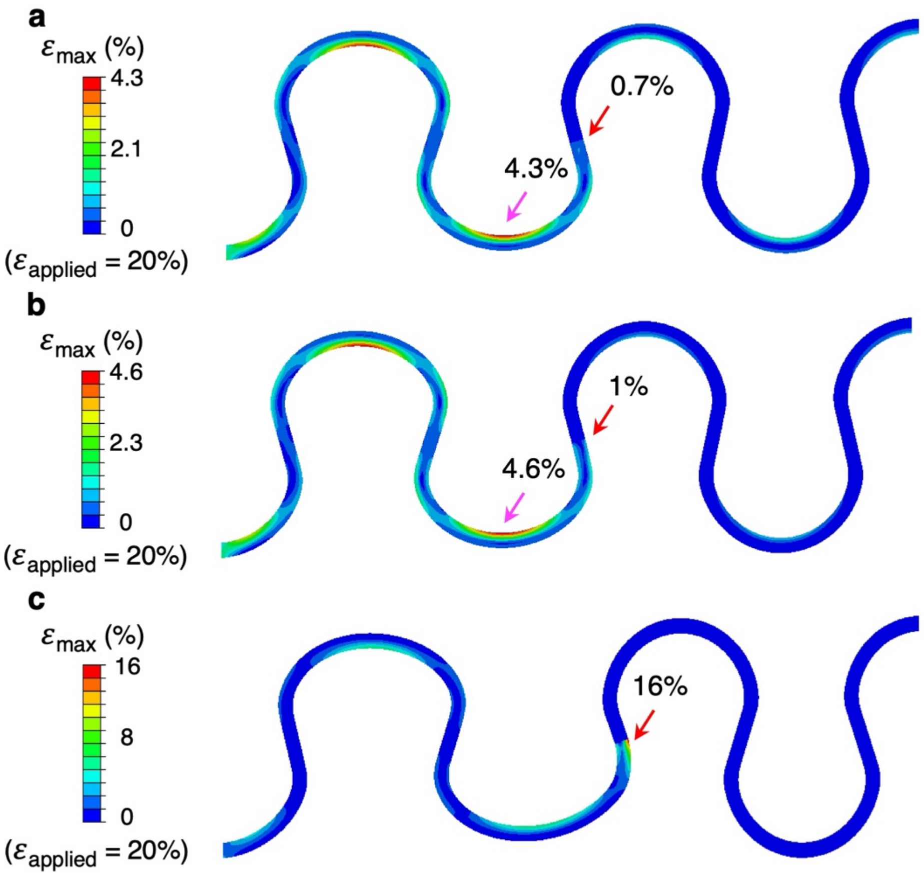

Supplementary Fig. 10. The maximum strain on HSPR (Arm) depending on stiffness ratios. The 300-nm-thin GET is connected to **a)** 750-nm-thin Au/PI, **b)** 13-µm-thick Au/PI, and **c)** 18-µm-thick Cu, which has the stiffness ratio of 7.7, 37.6, and 1596, respectively. Red arrows indicate the edge of Au/PI and pink arrows indicate the global maximum strain. The HSPRs are supported by 100-µm-thick Ecoflex, and it is not displayed in the simulation results.

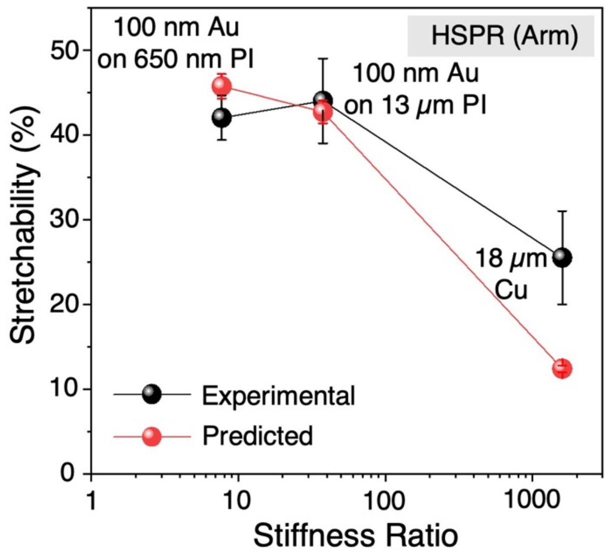

Supplementary Fig. 11. Stretchability of HSPR (Arm) with different stiffness ratios. Error bars represent the standard deviation.

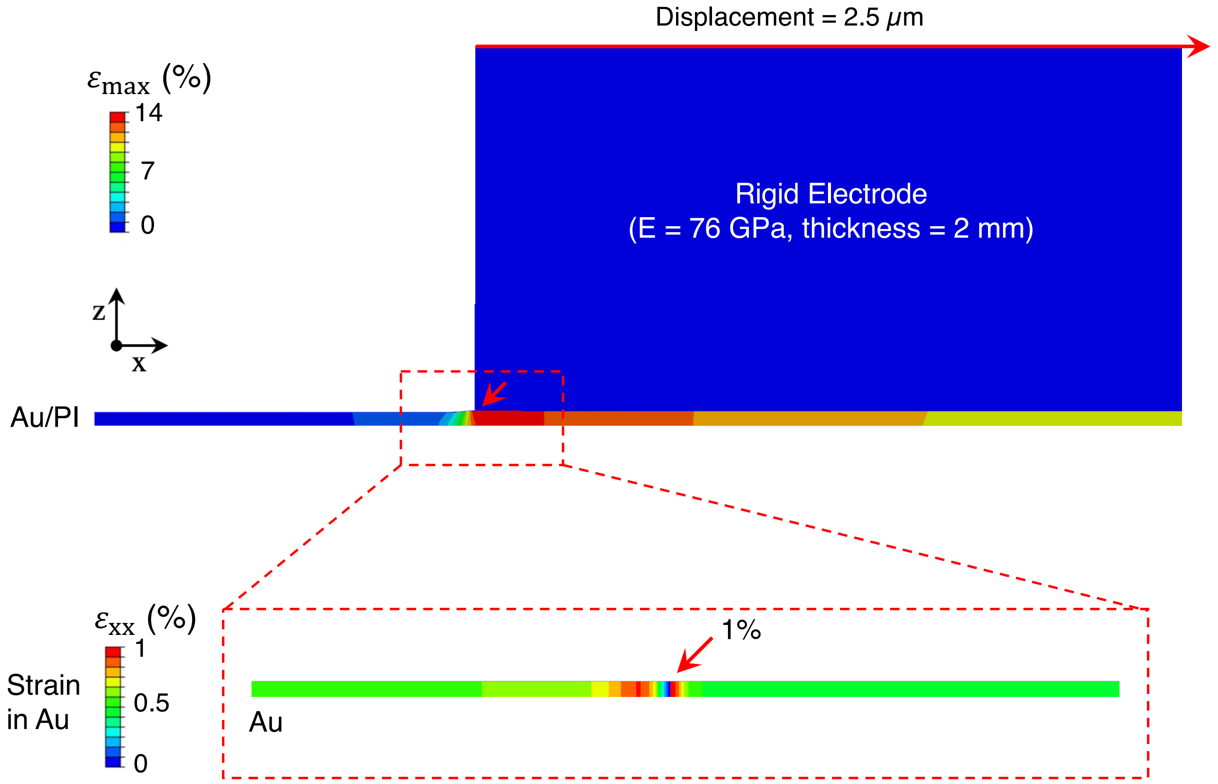

Supplementary Fig. 12. Maximum strain in Au without the soft interlayer (FEM).

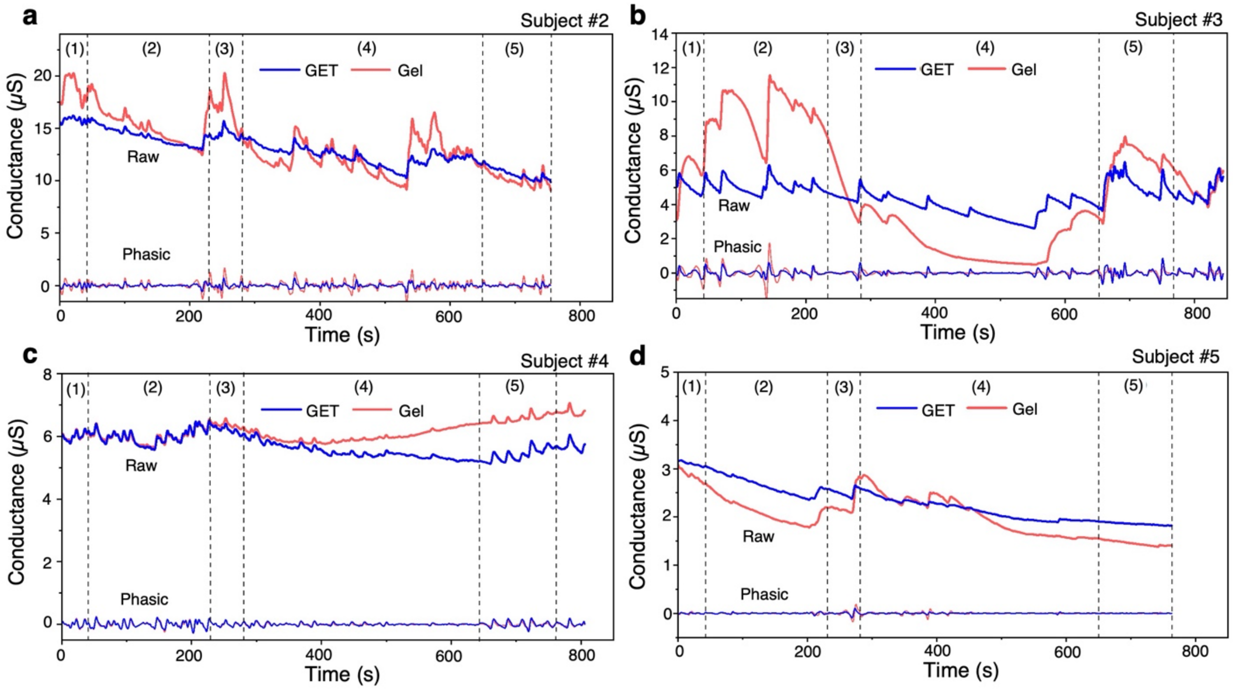

Supplementary Fig. 13. EDA measurement. **a-d)** EDA data from different human subjects (#2 - #5, respectively).

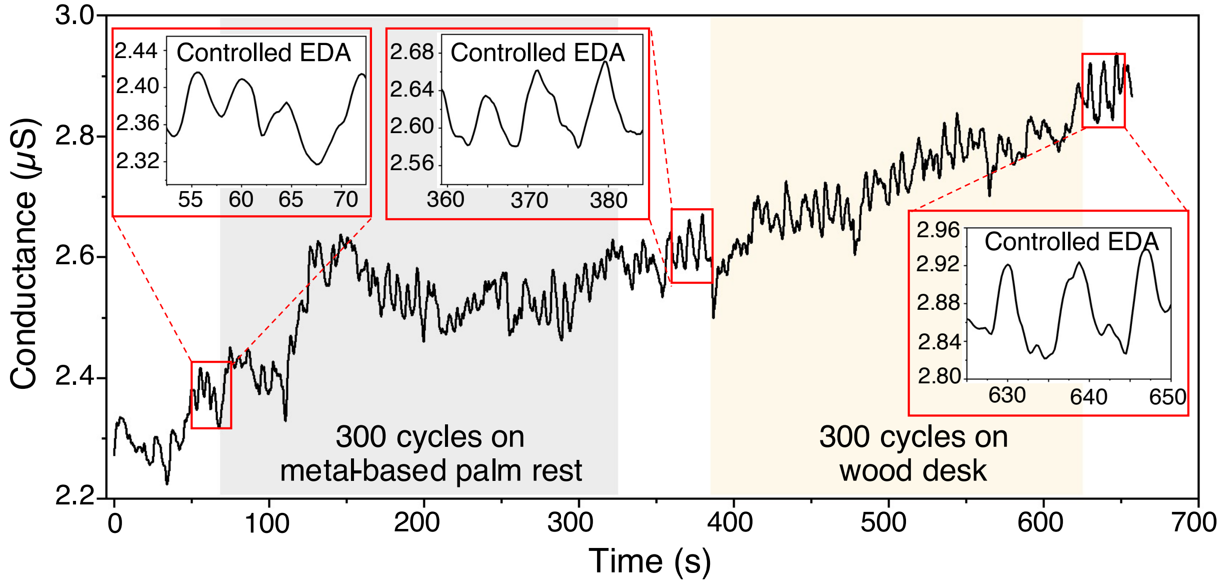

Supplementary Fig. 14. Friction test of GET-based EDA sensor. Conductance was measured over time while the GET-based EDA sensor was rubbed on the metallic palm rest of a keyboard (300 cycles) and a wood desk (300 cycles). Inset figures show the controlled EDA responses. The experimental setup and real-time data can be found in Supplementary Movie 1.

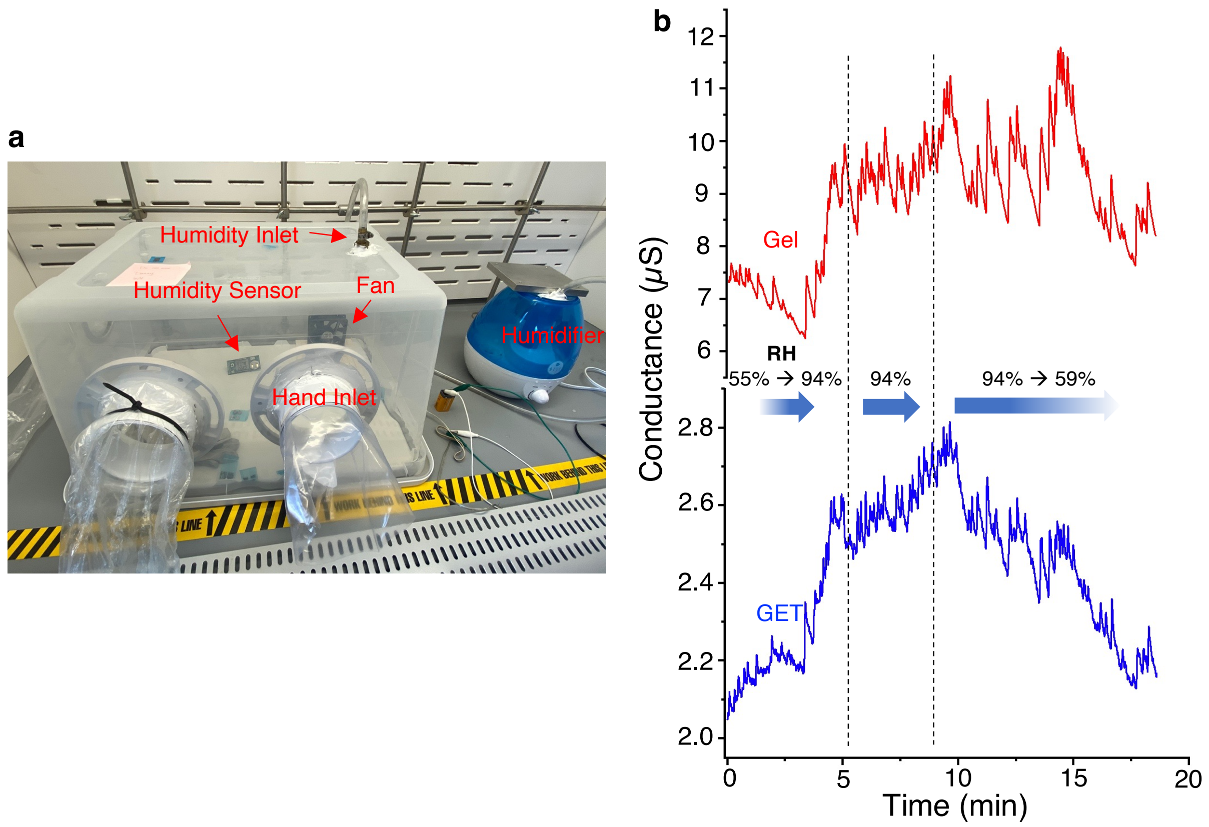

Supplementary Fig. 15. Environmental humidity effect on EDA responses. a) A homemade humidity chamber in which the EDA test was carried out. **b)** Total skin conductance measured by gel-based (red) and GET-based (blue) EDA sensors while the palm is placed in the humidity chamber for 20 min. Detailed experimental setup and real-time data can be found in Supplementary Movie 2.

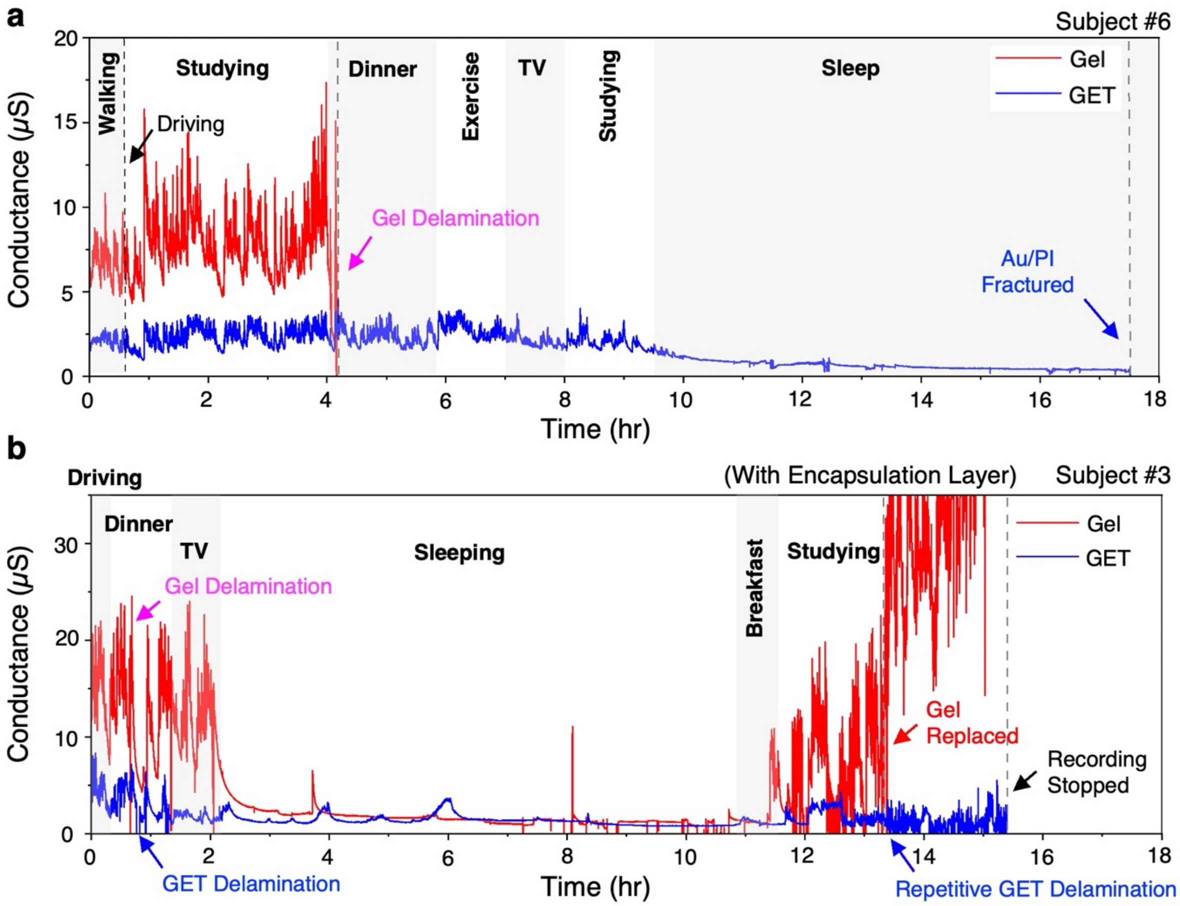

Supplementary Fig. 16. EDA long-term monitoring data. a) Long-term wearability test on subject #6. Ag/AgCl gel electrodes were not replaced after delamination. **b)** Another long-term wearability test. This time, GET was encapsulated by a 47-µm-thick overlay (Tegaderm, 3M).

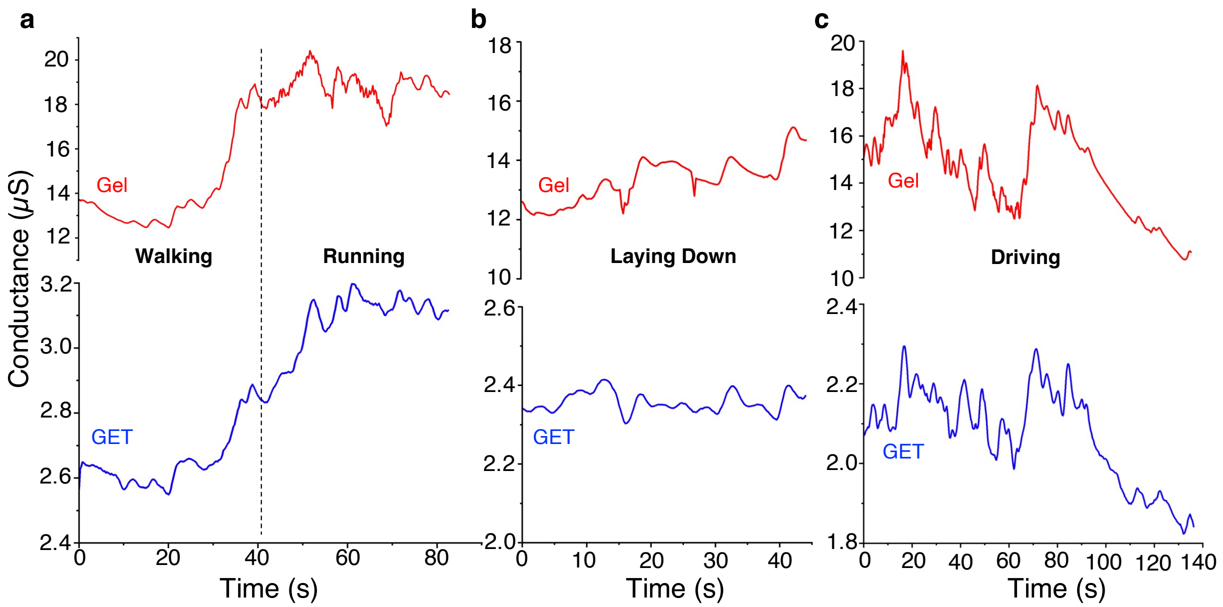

Supplementary Fig. 17. Additional EDA results during ambulatory activities. Both GET-based and gel-based EDA sensors are worn on the palm and EDA responses are monitored during **a)** walking and running, **b)** laying down, and **c)** driving. Detailed experimental setup and real-time data can be found in Supplementary Movie 3.

Supplementary Table 1. Survey of ultrathin wearable electrodes for their performance in monitoring biosignals. Same-sized electrodes were used for the comparison of the contact impedance.

Reference	Electrodes	Skin Contact Impedance Compared to Gel Electrodes (at 100 Hz)	Sensing Modality	Ambulatory Testing
10.1002/advs.201700771	PEDOT:PSS/EC	~120 kohms higher	EMG, EEG	No
10.1038/nnano.2017.125	Au nanomesh	~90 kohms higher	EMG	No
10.1002/smll.202000450	AgNWs/PDMS	~35 kohms lower	ECG, EMG	No
10.1021/acsami.0c04134	PEDOT:PSS/AgNWs	~50 kohms lower	ECG, EMG	No
10.1038/s41565-022-01145-w	GET	Not measured at 100 Hz	Blood pressure	No
This work	GET	~60 kohms lower	ECG, EMG, EEG, skin hydration, skin temperature, EDA	Yes

Supplementary Table 2. Index of the affective pictures used from the EmoMadrid database. Mean values are scaled from 0 (neutral) to 2 (maximum arousal) by EmoMadrid. Images from No. 1 – 10 are used for the session (4) and No. 11 is used for the session (5).

No.	EM Code	Mean	SD
1	506	0	0.43
2	736	1.75	0.46
3	453	1.15	0.59
4	710	1.86	0.35
5	728	1.8	0.4
6	411	0.83	0.56
7	597	1.65	0.51
8	158	0.02	0.41
9	361	0.94	0.55
10	56	0.01	0.46
11	618	1.84	0.37

Supplementary Table 3. EDA correlation score for human subject #1.

Session		Mean (Gel)	Mean (GET)	Mean Error (ME)	95% CI of ME	*p*-value	N
(2)	**Amplitude [μS]**	0.305	0.214	0.092	0.101	0.149	5
	$t_{peak}/T_{response}$	0.52	0.547	-0.027	0.024	0.098	5
	t_{rise} **[ms]**	1016	916	100	160	0.288	5
	$t_{rec, 50\%}$	644	620	24	101	0.666	5
	$t_{rec, 10\%}$	1242	1034	208	373	0.336	5
(4)	**Amplitude [μS]**	0.174	0.154	0.019	0.022	0.108	13
	$t_{peak}/T_{response}$	0.6	0.59	0.01	0.032	0.572	13
	t_{rise} **[ms]**	859	996	-137	204	0.212	13
	$t_{rec, 50\%}$	370	412	-42.3	38.9	0.055	13
	$t_{rec, 10\%}$	710	792	-81.5	91.8	0.108	13
(5)	**Amplitude [μS]**	0.321	0.229	0.092	0.066	0.033	7
	$t_{peak}/T_{response}$	0.527	0.568	-0.041	0.028	0.028	7
	t_{rise} **[ms]**	763	798	-35.7	59.8	0.287	7
	$t_{rec, 50\%}$	393	472	-78.6	125	0.265	7
	$t_{rec, 10\%}$	884	801	82.8	88.0	0.115	7

Supplementary Table 4. EDA correlation score for human subject #4.

Session		Mean (Gel)	Mean (GET)	Mean Error (ME)	95% CI of ME	*p*-value	N
(2)	**Amplitude [μS]**	0.697	0.410	0.287	0.115	0.04	3
	$t_{peak}/T_{response}$	0.458	0.568	-0.111	0.13	0.238	3
	t_{rise} **[ms]**	796.7	853.3	-56.7	219.8	0.664	3
	$t_{rec, 50\%}$	390	656.7	-266.7	455.79	0.370	3
	$t_{rec, 10\%}$	786.7	803.3	-16.7	313.26	0.926	3
(4)	**Amplitude [μS]**	0.572	0.248	0.324	0.169	<0.005	12
	$t_{peak}/T_{response}$	0.523	0.494	0.030	0.046	0.231	12
	t_{rise} **[ms]**	697.5	661.7	35.8	68.4	0.327	12
	$t_{rec, 50\%}$	445	544.2	-99.2	184.5	0.315	12
	$t_{rec, 10\%}$	779.2	970	-190.8	263.9	0.184	12
(5)	**Amplitude [μS]**	0.762	0.227	0.535	0.442	0.254	2
	$t_{peak}/T_{response}$	0.385	0.438	-0.052	0.238	0.742	2
	t_{rise} **[ms]**	840	725	115	29.4	0.083	2
	$t_{rec, 50\%}$	530	480	50	254.8	0.766	2
	$t_{rec, 10\%}$	1650	855	795	676.2	0.261	2

Supplementary Table 5. EDA correlation score for human subject #5.

Session		Mean (Gel)	Mean (GET)	Mean Error (ME)	95% CI of ME	*p*-value	N
**(2)**	**Amplitude [μS]**	0.115	0.105	0.01	0.028	0.49	8
	$t_{peak}/T_{response}$	0.572	0.574	-0.002	0.022	0.869	8
	t_{rise} **[ms]**	652.5	668.75	-16.25	89.28	0.732	8
	$t_{rec, 50\%}$	312.5	312.5	0	34.94	1	8
	$t_{rec, 10\%}$	630	607.5	22.5	41.21	0.32	8
**(4)**	**Amplitude [μS]**	0.074	0.114	-0.039	0.014	<0.005	10
	$t_{peak}/T_{response}$	0.479	0.465	0.015	0.045	0.533	10
	t_{rise} **[ms]**	1013	1015	-2	178.1	0.983	10
	$t_{rec, 50\%}$	532	840	-308	208.8	0.018	10
	$t_{rec, 10\%}$	1209	1721	-512	352.8	0.019	10
**(5)**	**Amplitude [μS]**	0.097	0.231	-0.134	0.026	0.01	3
	$t_{peak}/T_{response}$	0.536	0.525	0.011	0.059	0.749	3
	t_{rise} **[ms]**	910	1583.3	-673.3	1056.4	0.338	3
	$t_{rec, 50\%}$	466.7	980	-513.3	351.04	0.103	3
	$t_{rec, 10\%}$	960	1766.7	-806.7	492.3	0.085	3
