# Supplementary material for: Graphene e-tattoos for unobstructive ambulatory electrodermal activity sensing on the palm enabled by heterogeneous serpentine ribbons
Source: Nat Commun. 2022 Nov 3;13:6604. doi: 10.1038/s41467-022-34406-2 (PMC9633646; doi:10.1038/s41467-022-34406-2)
Supplement: Supplementary file 2 — Description of Additional Supplementary Files [file 41467_2022_34406_MOESM2_ESM.docx]

**Description of Additional Supplementary Files**

**File Name: Supplementary Movie 1
Description:** Friction tests of GET-based EDA sensor worn on the palm subjected to cyclic rubbing on a laptop and a wood desk.

**File Name: Supplementary Movie 2
Description:** Environmental humidity effects on EDA responses measured by GETbased EDA sensor and gel-based EDA sensor.

**File Name: Supplementary Movie 3
Description:** Simultaneous ambulatory tests on both GET and gel based EDA sensors during 1) walking and running, 2) sleeping, and 3) driving.
